# Correction: Lytic Gene Expression Is Frequent in HSV-1 Latent Infection and Correlates with the Engagement of a Cell-Intrinsic Transcriptional Response

**DOI:** 10.1371/journal.ppat.1004361

**Published:** 2014-08-08

**Authors:** The PLOS Pathogens Staff

## Notice of Republication

This article was republished on July 30^th^, 2014, to correct a corrupted Figure 5 in the original PDF download. Please download this article again to view the correct version. The originally published, uncorrected article and the republished, corrected article are provided here for reference.

## Supporting Information

File S1 Originally published, uncorrected article.
(PDF)

File S2 Republished corrected article.
(PDF)
