# Supplementary figures and images for: Lytic Gene Expression Is Frequent in HSV-1 Latent Infection and Correlates with the Engagement of a Cell-Intrinsic Transcriptional Response
Source: PLoS Pathog. 2014 Jul 24;10(7):e1004237. doi: 10.1371/journal.ppat.1004237 (PMC4110040; doi:10.1371/journal.ppat.1004237)

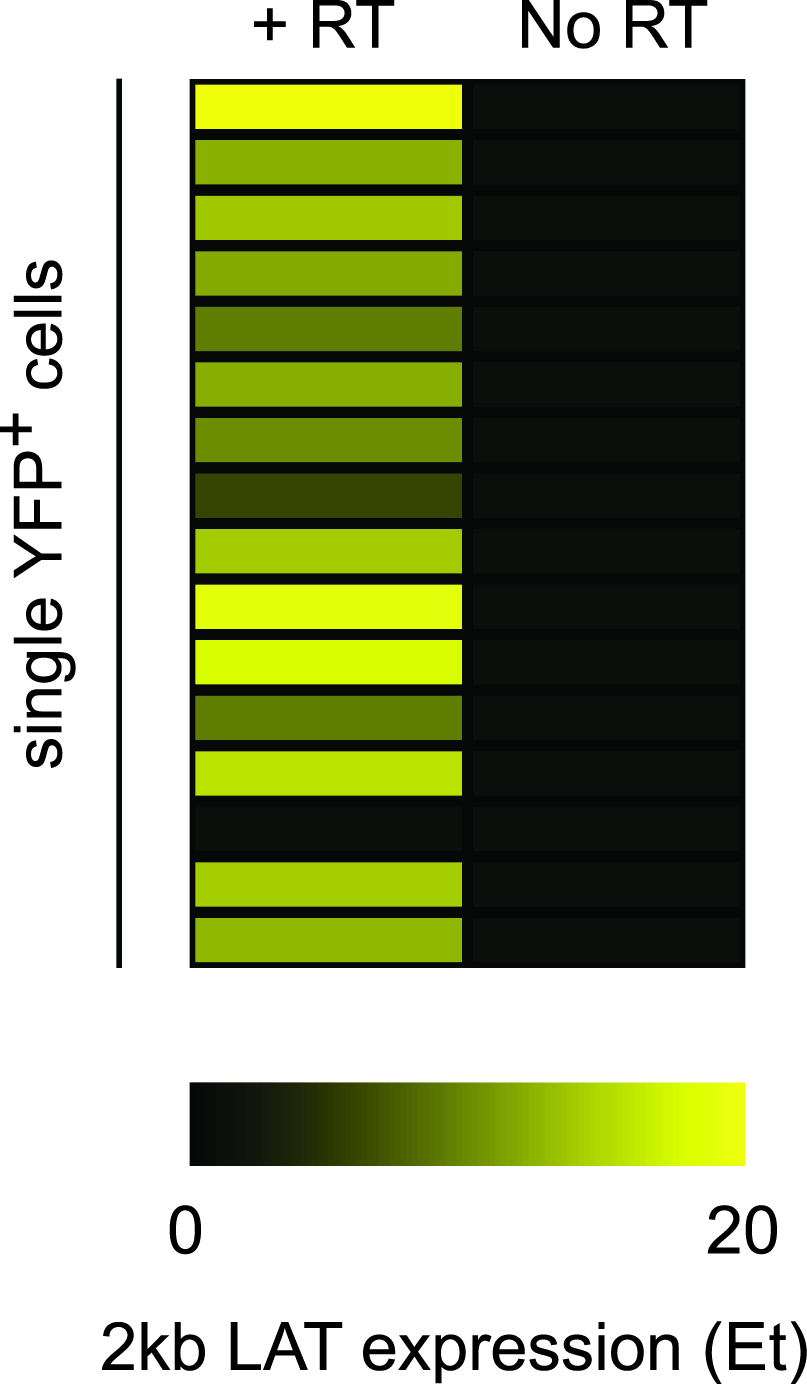

Supplement: Figure S1 — Single cells are free of contaminating viral DNA. Heatmap showing detection of 2 kb LAT in standard (+RT) and sham (No RT) reverse transcribed RNA from a set of 16 YFP+ neurons. (TIF) [file ppat.1004237.s001.tif]

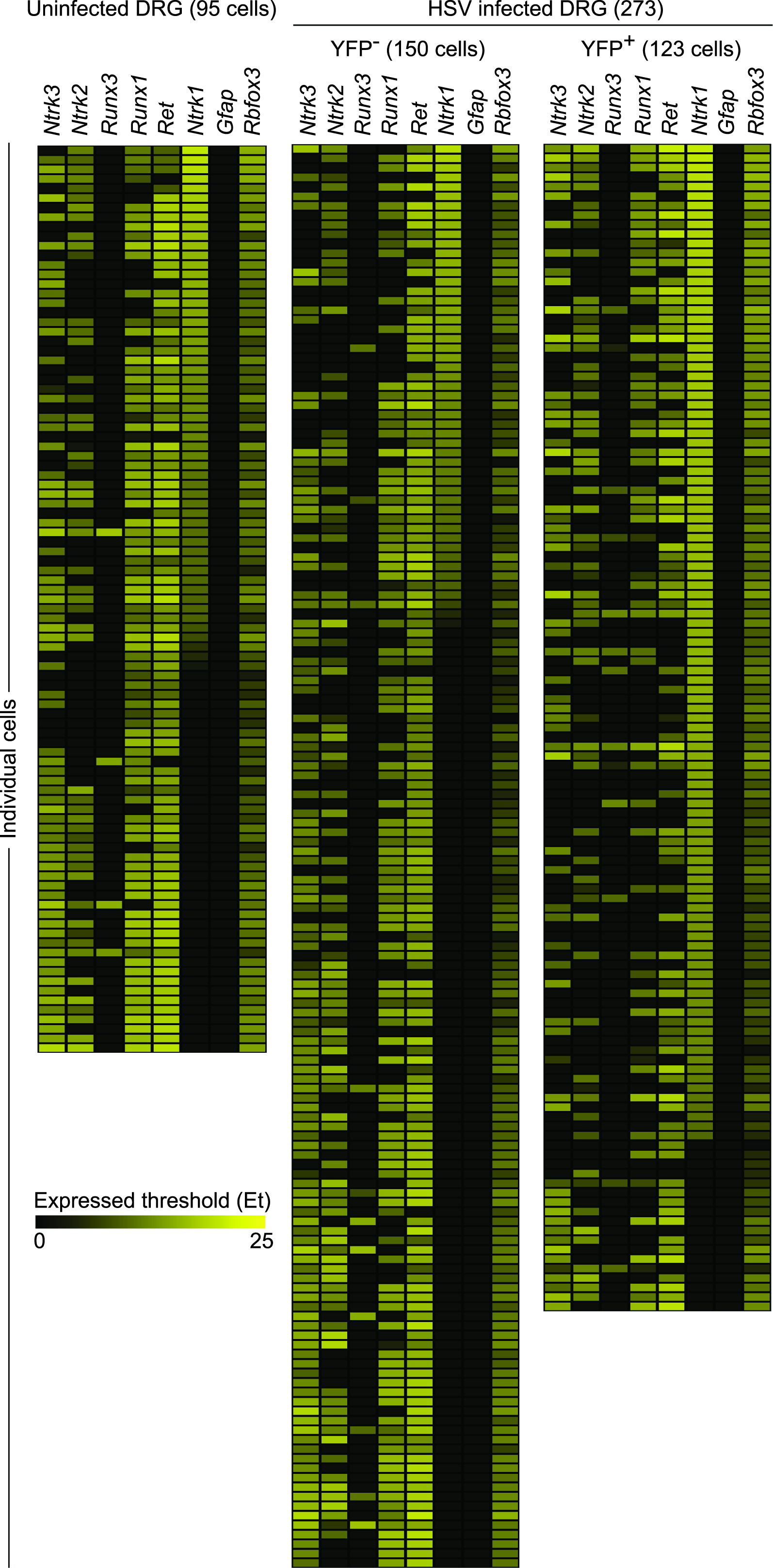

Supplement: Figure S2 — Heatmap showing expression of neuronal-related genes in single neurons (Rbfox3 + Gfap −) determined using quantitative RT-PCR. Data from main single cell experiments. (TIF) [file ppat.1004237.s002.tif]

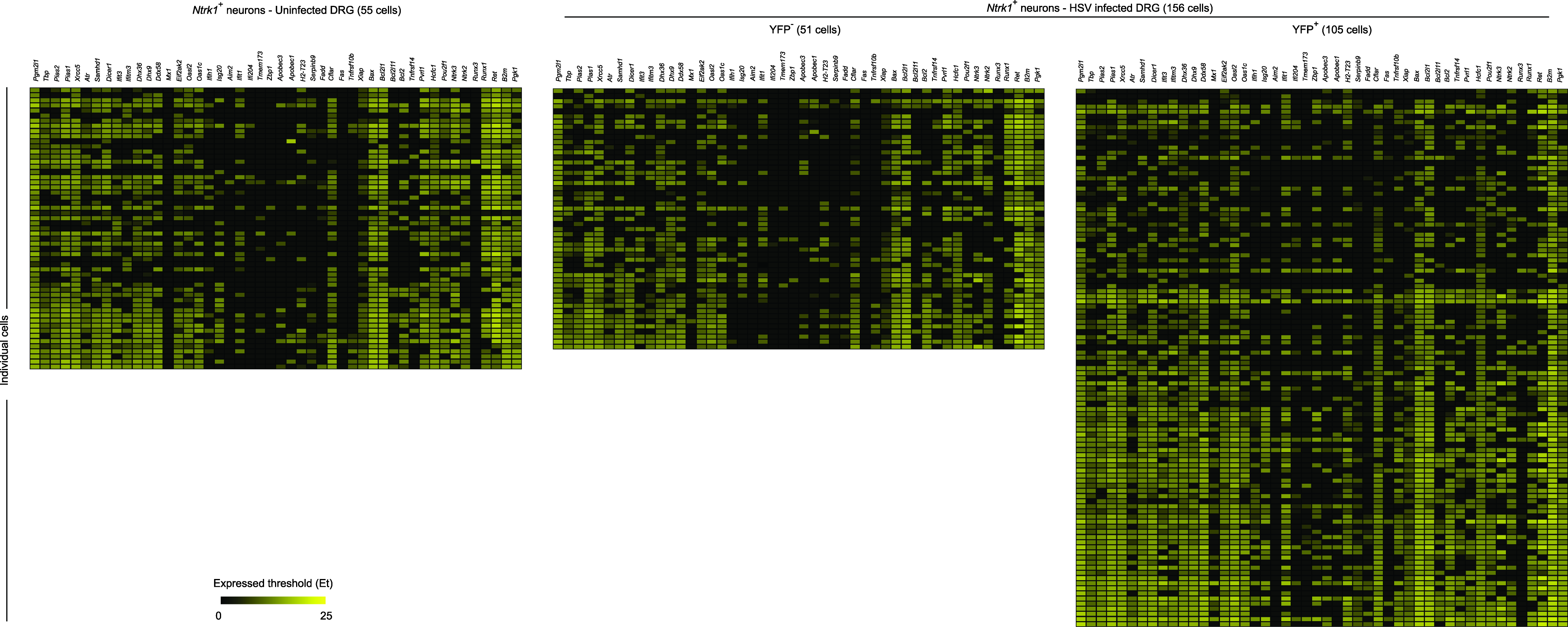

Supplement: Figure S3 — Heatmap showing 48 cellular genes in single Ntrk1 + neurons determined using quantitative RT-PCR. Data from main single cell experiments. (TIF) [file ppat.1004237.s003.tif]

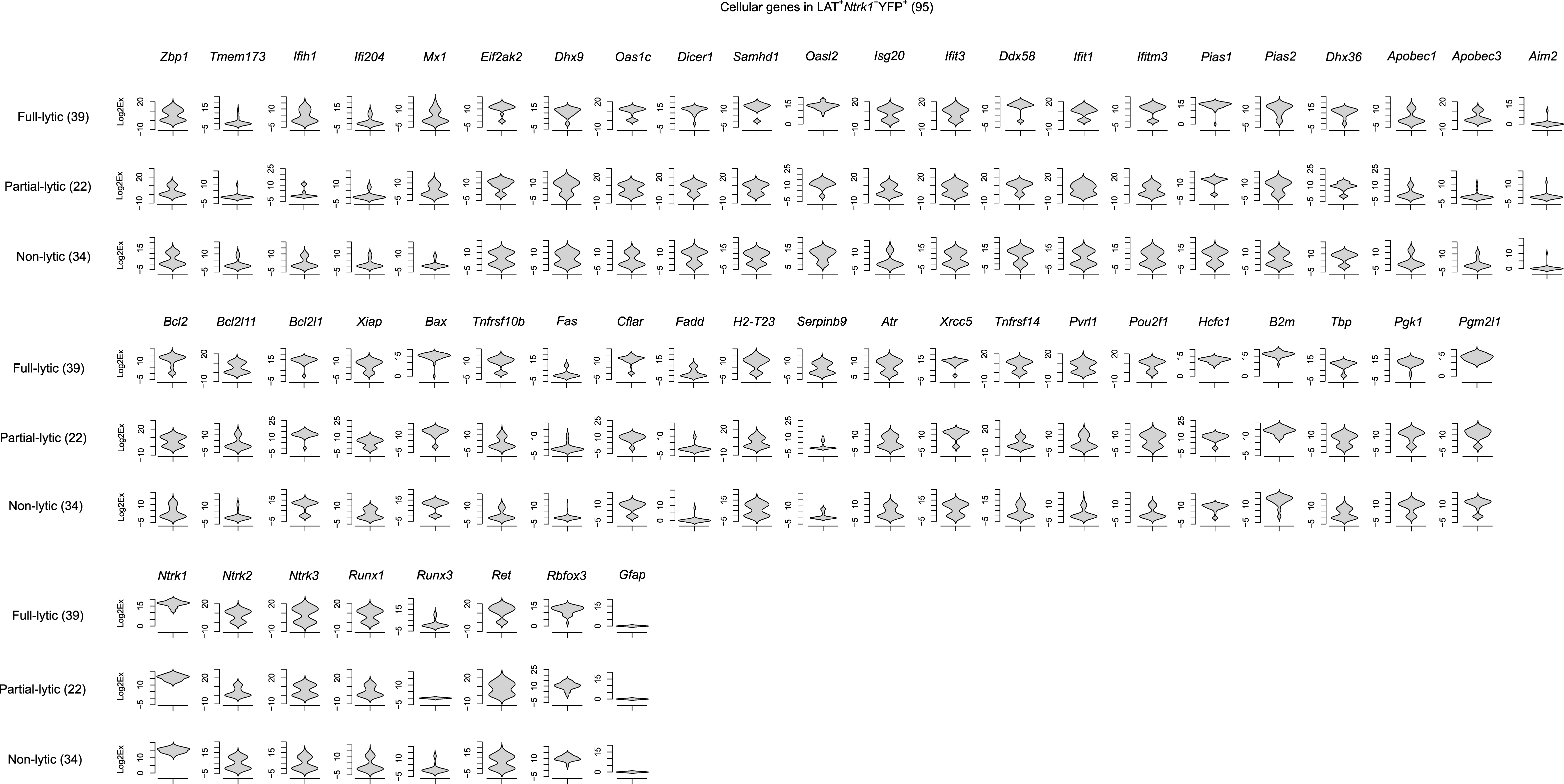

Supplement: Figure S4 — Violin plot representation of 48 cellular transcripts in LAT+ Ntrk1 +YFP+ neurons, categorized based on their lytic gene expression profiles. Data from main single cell experiments. (TIF) [file ppat.1004237.s004.tif]
