# Supplementary material for: Lytic Gene Expression Is Frequent in HSV-1 Latent Infection and Correlates with the Engagement of a Cell-Intrinsic Transcriptional Response
Source: PLoS Pathog. 2014 Jul 24;10(7):e1004237. doi: 10.1371/journal.ppat.1004237 (PMC4110040; doi:10.1371/journal.ppat.1004237)
Supplement: Table S1 — Numbers of neurons excluded from gene transcripts analysis. (DOCX) [file ppat.1004237.s005.docx]

**Table S1.** Numbers of neurons excluded from final transcriptional analyses

|  | **Groups of neurons** | | |  |
| --- | --- | --- | --- | --- |
|  | **Uninfected** | **YFP^-^** | **YFP^+^** | **Total** |
| **Total neurons** | 136 | 256 | 205 | 597 |
|  |  |  |  |  |
| **Exclusion or error type** |  |  |  |  |
| *Rbfox3* negative | - 29 | - 35 | - 56 |  |
| *Gfap* positive | - 1 | - 6 | - 22 |  |
| HSV transcripts in uninfected | - 9 | - 59 | n/a |  |
| <10% cellular genes expressed | - 2 | - 6 | - 4 |  |
| *Ntrk1* negative | - 40 | - 99 | - 18 |  |
| LAT negative | n/a | n/a | - 10 |  |
|  |  |  |  |  |
| **Neurons remaining for analysis** | 55 | 51 | 95 | 201 |

n/a. Not applicable
