# Supplementary material for: Lytic Gene Expression Is Frequent in HSV-1 Latent Infection and Correlates with the Engagement of a Cell-Intrinsic Transcriptional Response
Source: PLoS Pathog. 2014 Jul 24;10(7):e1004237. doi: 10.1371/journal.ppat.1004237 (PMC4110040; doi:10.1371/journal.ppat.1004237)
Supplement: Table S2 — Ratio of mean gene expression levels (Et) from comparisons within all, Ntrk1 + and LAT+ Ntrk1 +YFP+ neurons. (DOCX) [file ppat.1004237.s006.docx]

**Table S2.** Ratio of mean of gene expression levels (Et) from comparisons within all, *Ntrk1*^+^ and LAT^+^*Ntrk1*^+^YFP^+^ neurons.

|  | All neurons | | | *Ntrk1*^+^ neurons | | | LAT^+^*Ntrk1*^+^YFP^+^ neurons | | |
| --- | --- | --- | --- | --- | --- | --- | --- | --- | --- |
|  | YFP^+^ **vs.** YFP^-^ | YFP^+^ **vs.** Uninfected | YFP^-^ **vs.** Uninfected | YFP^+^ **vs**. YFP^-^ | YFP^+^ **vs**. Uninfected | YFP^-^ **vs**. Uninfected | Full-lytic **vs**. Partial-lytic | Full-lytic **vs**. non-lytic | Partial-lytic **vs**. non-lytic |
| *Pgm2l1* | 0.8930 | 0.8741 | 0.9788 | 0.9447 | 0.8605 | 0.9108 | 1.3277 | 1.4598 | 1.0995 |
| *Tbp* | 1.1778 | 0.9540 | 0.8100 | 1.2895 | 0.9127 | 0.7078 | 1.5850 | 2.7090 | 1.7092 |
| *Pias2* | 1.1464 | 0.9298 | 0.8111 | 1.3389 | 0.9111 | 0.6805 | 1.3346 | 1.9464 | 1.4584 |
| *Pias1* | 1.0567 | 0.9379 | 0.8876 | 1.1510 | 0.9301 | 0.8081 | 1.2683 | 1.8379 | 1.4491 |
| *Xrcc5* | 0.9544 | 0.8602 | 0.9013 | 1.0151 | 0.8615 | 0.8487 | 1.2062 | 1.7338 | 1.4373 |
| *Atr* | 1.2113 | 0.9644 | 0.7961 | 1.5210 | 0.9175 | 0.6032 | 1.5882 | 1.7564 | 1.1059 |
| *Samhd1* | 1.2009 | 1.0973 | 0.9138 | 1.3362 | 1.0293 | 0.7703 | 1.4791 | 1.6670 | 1.1270 |
| *Dicer1* | 1.0389 | 0.9308 | 0.8960 | 1.1793 | 0.9433 | 0.7999 | 1.4949 | 1.7984 | 1.2030 |
| *Ifit3* | 1.5897 | 1.3545 | 0.8520 | 1.6375 | 1.1612 | 0.7091 | 1.2617 | 1.4528 | 1.1514 |
| *Ifitm3* | 1.8969 | 1.5013 | 0.7914 | 2.0600 | 1.2830 | 0.6228 | 1.8471 | 1.4950 | 0.8094 |
| *Dhx36* | 1.1116 | 1.0042 | 0.9034 | 1.1169 | 1.0678 | 0.9560 | 1.2247 | 1.4367 | 1.1732 |
| *Dhx9* | 1.0790 | 0.9891 | 0.9167 | 1.0705 | 0.9472 | 0.8848 | 1.4160 | 1.9034 | 1.3442 |
| *Ddx58* | 1.4798 | 1.6491 | 1.1144 | 1.5635 | 1.3175 | 0.8427 | 1.2866 | 1.4929 | 1.1603 |
| *Mx1* | 9.2922 | - | - | 13.9231 | - | - | 1.1050 | 3.4722 | 3.1423 |
| *Eif2ak2* | 1.2865 | 1.2167 | 0.9458 | 1.2720 | 1.1884 | 0.9343 | 1.2382 | 1.9592 | 1.5823 |
| *Oasl2* | 1.5813 | 1.8840 | 1.1914 | 1.5753 | 1.7515 | 1.1118 | 1.2518 | 1.5609 | 1.2469 |
| *Oas1c* | 0.9532 | 1.0053 | 1.0547 | 1.1474 | 0.9272 | 0.8082 | 1.4736 | 2.2222 | 1.5080 |
| *Ifih1* | 1.9461 | 1.8143 | 0.9323 | 2.5902 | 1.5583 | 0.6016 | 1.8330 | 1.6962 | 0.9254 |
| *Isg20* | 1.8187 | 1.2197 | 0.6706 | 1.7215 | 1.2597 | 0.7317 | 1.6489 | 2.8195 | 1.7099 |
| *Aim2* | 1.8257 | 3.2680 | 1.7900 | - | 2.2163 | 0.0000 | 0.9959 | 1.7431 | 1.7503 |
| *Ifit1* | 1.8626 | 2.0605 | 1.1063 | 1.9104 | 1.8405 | 0.9634 | 1.4131 | 1.5643 | 1.1070 |
| *Ifi204* | 9.1906 | 5.7830 | 0.6292 | 19.6888 | 3.4710 | 0.1763 | 1.8513 | 1.2030 | 0.6498 |
| *Tmem173* | 1.6196 | 1.2254 | 0.7566 | 1.9497 | 1.2192 | 0.6254 | 2.9458 | 0.7989 | 0.2712 |
| *Zbp1* | 4.8730 | 20.0684 | 4.1183 | 11.1173 | 12.4970 | 1.1241 | 1.7785 | 1.4665 | 0.8246 |
| *Apobec3* | 1.7487 | 1.9570 | 1.1191 | 1.0418 | 1.6611 | 1.5945 | 4.6445 | 1.9583 | 0.4217 |
| *Apobec1* | 2.1386 | 2.3048 | 1.0777 | 1.8893 | 1.6537 | 0.8753 | 1.4725 | 1.1841 | 0.8041 |
| *H2-T23* | 1.7044 | 2.3076 | 1.3539 | 2.2964 | 2.0186 | 0.8790 | 1.8422 | 1.4641 | 0.7948 |
| *Serpinb9* | 2.2305 | 1.5900 | 0.7129 | 3.5754 | 1.6035 | 0.4485 | 2.2864 | 2.5240 | 1.1039 |
| *Fadd* | 1.3249 | 1.2877 | 0.9719 | 1.9014 | 1.3200 | 0.6942 | 3.1861 | 5.5610 | 1.7454 |
| *Cflar* | 1.3543 | 1.1672 | 0.8619 | 1.4433 | 1.1176 | 0.7743 | 1.1678 | 1.3667 | 1.1704 |
| *Fas* | 7.5812 | 4.0545 | 0.5348 | - | 5.3662 | 0.0000 | 1.3431 | 2.0695 | 1.5408 |
| *Tnfrsf10b* | 5.8275 | 4.2172 | 0.7237 | 3.4072 | 3.9288 | 1.1531 | 2.9224 | 3.6858 | 1.2612 |
| *Xiap* | 1.1567 | 0.8621 | 0.7453 | 1.0790 | 0.8930 | 0.8276 | 1.2874 | 2.1031 | 1.6336 |
| *Bax* | 1.0821 | 1.0002 | 0.9242 | 1.1722 | 0.9709 | 0.8283 | 1.2189 | 1.4924 | 1.2244 |
| *Bcl2l1* | 0.9748 | 0.8922 | 0.9153 | 1.0640 | 0.8926 | 0.8389 | 1.1205 | 1.4650 | 1.3074 |
| *Bcl2l11* | 3.1163 | 1.8054 | 0.5793 | 2.1324 | 1.4166 | 0.6643 | 2.0411 | 4.1664 | 2.0413 |
| *Bcl2* | 1.2809 | 1.2907 | 1.0076 | 1.1842 | 1.4429 | 1.2184 | 1.6847 | 3.0631 | 1.8182 |
| *Tnfrsf14* | 3.2654 | 2.2279 | 0.6823 | 1.9628 | 1.5877 | 0.8089 | 2.2814 | 2.0626 | 0.9041 |
| *Pvrl1* | 0.5775 | 0.5084 | 0.8804 | 0.6421 | 0.4886 | 0.7610 | 1.7611 | 2.7730 | 1.5746 |
| *Hcfc1* | 1.0340 | 1.0004 | 0.9675 | 1.1179 | 0.9714 | 0.8689 | 1.2496 | 1.3737 | 1.0994 |
| *Pou2f1* | 1.0760 | 0.8892 | 0.8265 | 1.2737 | 0.9013 | 0.7077 | 1.3585 | 3.3791 | 2.4873 |
| *Ntrk3* | 0.7156 | 0.6870 | 0.9601 | 1.0490 | 0.7212 | 0.6875 | 1.4642 | 1.8953 | 1.2944 |
| *Ntrk2* | 0.7589 | 0.8935 | 1.1774 | 0.9679 | 0.8669 | 0.8957 | 2.2206 | 1.6205 | 0.7298 |
| *Runx3* | 1.1239 | 1.5943 | 1.4186 | 1.7103 | 3.2536 | 1.9023 | - | 1.1908 | 0.0000 |
| *Runx1* | 0.6051 | 0.4696 | 0.7760 | 0.6931 | 0.5040 | 0.7272 | 1.8682 | 2.5074 | 1.3422 |
| *Ret* | 0.6719 | 0.6410 | 0.9539 | 0.6718 | 0.6316 | 0.9401 | 1.5222 | 1.9171 | 1.2594 |
| *Ntrk1* | 2.9886 | 1.7609 | 0.5892 | - | - | - | - | - | - |
| *B2m* | 1.1090 | 1.2745 | 1.1492 | 1.1192 | 1.2253 | 1.0948 | 1.1572 | 1.2340 | 1.0663 |
| *Pgk1* | 0.9744 | 0.9669 | 0.9924 | 1.0382 | 0.9472 | 0.9124 | 1.3014 | 1.5762 | 1.2112 |
