# Supplementary material for: Lytic Gene Expression Is Frequent in HSV-1 Latent Infection and Correlates with the Engagement of a Cell-Intrinsic Transcriptional Response
Source: PLoS Pathog. 2014 Jul 24;10(7):e1004237. doi: 10.1371/journal.ppat.1004237 (PMC4110040; doi:10.1371/journal.ppat.1004237)
Supplement: Table S3 — p values for the gene expression levels from comparisons within all, Ntrk1 + and LAT+ Ntrk1 +YFP+ neurons. (DOCX) [file ppat.1004237.s007.docx]

**Table S3.** *p* values for the gene expression levels from comparisons within all, *Ntrk1*^+^ and LAT^+^*Ntrk1*^+^YFP^+^ neurons.

|  | All neurons | | | *Ntrk1*^+^ neurons | | | LAT^+^*Ntrk1*^+^YFP^+^ neurons | | |
| --- | --- | --- | --- | --- | --- | --- | --- | --- | --- |
|  | YFP^+^ **vs.** YFP^-^ | YFP^+^ **vs.** Uninfected | YFP^-^ **vs.** Uninfected | YFP^+^ **vs**. YFP^-^ | YFP^+^ **vs**. Uninfected | YFP^-^ **vs**. Uninfected | Full-lytic **vs**. Partial-lytic | Full-lytic **vs**. non-lytic | Partial-lytic **vs**. non-lytic |
| *Pgm2l1* | **0.0113** | **0.0017** | 0.1664 | 0.4143 | **0.0085** | 0.0756 | **0.0047** | **0.0000** | 0.4595 |
| *Tbp* | 0.0937 | 0.7937 | 0.0493 | 0.0720 | 0.5160 | 0.0192 | **0.0006** | **0.0000** | 0.0293 |
| *Pias2* | 0.1340 | 0.4183 | 0.0173 | 0.0330 | 0.4862 | **0.0072** | 0.0220 | **0.0001** | 0.1152 |
| *Pias1* | 0.1024 | 0.7598 | 0.0486 | 0.0403 | 0.6100 | **0.0157** | **0.0003** | **0.0000** | **0.0082** |
| *Xrcc5* | 0.8915 | 0.1354 | 0.0649 | 0.3355 | 0.1497 | **0.0130** | **0.0100** | **0.0000** | 0.0232 |
| *Atr* | 0.1802 | 0.7696 | 0.1258 | 0.0535 | 0.5923 | 0.0359 | 0.0536 | **0.0087** | 0.7177 |
| *Samhd1* | **0.0099** | 0.4102 | 0.1387 | 0.0173 | 0.8572 | 0.0319 | **0.0114** | **0.0000** | 0.4515 |
| *Dicer1* | 0.3222 | 0.3835 | 0.0294 | 0.1298 | 0.4888 | 0.0209 | **0.0007** | **0.0000** | 0.1789 |
| *Ifit3* | **0.0005** | 0.0418 | 0.2266 | **0.0084** | 0.3518 | 0.0852 | 0.2927 | 0.1183 | 0.6226 |
| *Ifitm3* | **0.0000** | **0.0030** | 0.1968 | **0.0002** | 0.0796 | 0.0393 | **0.0043** | 0.0407 | 0.4109 |
| *Dhx36* | 0.1634 | 0.9463 | 0.1123 | 0.2879 | 0.8190 | 0.3456 | **0.0146** | **0.0001** | 0.1866 |
| *Dhx9* | 0.2821 | 0.9033 | 0.2287 | 0.3740 | 0.6794 | 0.1611 | **0.0148** | **0.0000** | 0.1764 |
| *Ddx58* | **0.0000** | **0.0000** | 0.6035 | **0.0000** | **0.0046** | 0.1894 | **0.0111** | **0.0002** | 0.5088 |
| *Mx1* | **0.0000** | **0.0000** | 0.0728 | **0.0002** | **0.0000** | 0.2990 | 0.8781 | **0.0118** | 0.0188 |
| *Eif2ak2* | **0.0017** | 0.0262 | 0.5499 | **0.0151** | 0.0999 | 0.4400 | 0.0524 | **0.0000** | 0.0191 |
| *Oasl2* | **0.0000** | **0.0000** | 0.1519 | **0.0000** | **0.0000** | 0.4897 | **0.0052** | **0.0000** | 0.1842 |
| *Oas1c* | 0.8719 | 0.8151 | 0.1519 | 0.3217 | 0.8178 | 0.2605 | 0.0392 | **0.0000** | 0.0765 |
| *Ifih1* | **0.0079** | 0.0208 | 0.9888 | 0.0214 | 0.1105 | 0.4646 | 0.1338 | 0.1171 | 1.0000 |
| *Isg20* | **0.0011** | 0.2982 | 0.0536 | 0.0186 | 0.2789 | 0.2339 | 0.0376 | **0.0008** | 0.1753 |
| *Aim2* | 0.4942 | 0.2758 | 0.5723 | 0.1593 | 0.4782 | 0.3356 | 0.9521 | 0.6411 | 0.7345 |
| *Ifit1* | **0.0000** | **0.0000** | 0.6192 | **0.0005** | **0.0005** | 0.8728 | 0.0471 | 0.0495 | 0.6990 |
| *Ifi204* | **0.0000** | **0.0005** | 0.7976 | **0.0030** | 0.0202 | 0.3334 | 0.3302 | 0.7253 | 0.4896 |
| *Tmem173* | 0.1792 | 0.5513 | 0.5407 | 0.2158 | 0.7516 | 0.3844 | 0.2169 | 0.7448 | 0.1611 |
| *Zbp1* | **0.0000** | **0.0000** | 0.0534 | **0.0000** | **0.0000** | 0.9237 | 0.1016 | 0.2072 | 0.6738 |
| *Apobec3* | 0.0817 | 0.0631 | 0.6645 | 0.9526 | 0.2341 | 0.2663 | 0.0247 | 0.1321 | 0.2377 |
| *Apobec1* | **0.0153** | **0.0118** | 0.5980 | 0.1727 | 0.1330 | 0.9016 | 0.4847 | 0.8075 | 0.7167 |
| *H2-T23* | **0.0002** | **0.0000** | 0.1521 | **0.0002** | **0.0007** | 0.5386 | **0.0093** | 0.0591 | 0.3734 |
| *Serpinb9* | **0.0005** | 0.0860 | 0.1408 | **0.0008** | 0.0972 | 0.0709 | 0.0219 | **0.0050** | 0.9832 |
| *Fadd* | 0.4464 | 0.4452 | 0.9195 | 0.1787 | 0.5248 | 0.4741 | 0.0250 | **0.0013** | 0.5989 |
| *Cflar* | **0.0002** | 0.0965 | 0.0810 | **0.0012** | 0.3443 | 0.0435 | 0.0207 | **0.0002** | 0.2745 |
| *Fas* | **0.0024** | **0.0161** | 0.9368 | **0.0090** | 0.0276 | 0.3356 | 0.6580 | 0.2591 | 0.5632 |
| *Tnfrsf10b* | **0.0000** | **0.0000** | 0.2118 | **0.0001** | **0.0000** | 0.9958 | **0.0001** | **0.0000** | 0.5916 |
| *Xiap* | 0.2267 | 0.1851 | **0.0071** | 0.5506 | 0.4103 | 0.1771 | 0.0478 | **0.0002** | 0.0604 |
| *Bax* | **0.0101** | 0.9248 | **0.0053** | **0.0024** | 0.8927 | **0.0013** | **0.0017** | **0.0000** | 0.0575 |
| *Bcl2l1* | 0.7949 | 0.0896 | 0.0511 | 0.6812 | 0.1234 | 0.0348 | **0.0107** | **0.0000** | 0.0575 |
| *Bcl2l11* | **0.0001** | 0.0273 | 0.1585 | 0.0344 | 0.2121 | 0.3817 | 0.0609 | **0.0010** | 0.2705 |
| *Bcl2* | 0.0333 | 0.0493 | 0.9822 | 0.2335 | 0.0271 | 0.3168 | **0.0009** | **0.0000** | 0.0696 |
| *Tnfrsf14* | **0.0000** | **0.0004** | 0.2042 | **0.0092** | 0.0395 | 0.5463 | **0.0062** | **0.0044** | 0.8268 |
| *Pvrl1* | **0.0000** | **0.0000** | 0.0281 | **0.0134** | **0.0000** | 0.0273 | 0.0652 | **0.0009** | 0.2928 |
| *Hcfc1* | 0.4158 | 0.9991 | 0.4326 | 0.1828 | 0.7862 | 0.1574 | **0.0021** | **0.0000** | 0.1636 |
| *Pou2f1* | 0.6350 | 0.3195 | 0.1464 | 0.2126 | 0.5252 | 0.1040 | 0.0400 | **0.0000** | **0.0051** |
| *Ntrk3* | **0.0069** | **0.0012** | 0.1560 | 0.7383 | 0.0326 | 0.0262 | 0.0593 | **0.0045** | 0.3202 |
| *Ntrk2* | 0.0184 | 0.4099 | 0.1581 | 0.8919 | 0.3402 | 0.3191 | **0.0103** | 0.0194 | 0.5393 |
| *Runx3* | 0.3988 | 0.1076 | 0.3486 | 0.3467 | 0.0544 | 0.2878 | 0.0550 | 0.7391 | 0.0983 |
| *Runx1* | **0.0000** | **0.0000** | **0.0007** | **0.0138** | **0.0000** | **0.0092** | 0.0220 | **0.0005** | 0.4036 |
| *Ret* | **0.0000** | **0.0000** | 0.1408 | **0.0001** | **0.0000** | 0.3932 | 0.0223 | **0.0001** | 0.3216 |
| *Ntrk1* | **0.0000** | **0.0000** | **0.0006** | Not tested | Not tested | Not tested | Not tested | Not tested | Not tested |
| *B2m* | **0.0000** | **0.0000** | 0.0229 | **0.0003** | **0.0000** | 0.2563 | **0.0080** | **0.0003** | 0.8932 |
| *Pgk1* | 0.6499 | 0.4744 | 0.6518 | 0.6770 | 0.4264 | 0.1985 | 0.0283 | **0.0000** | 0.0613 |

**Bold** values are considered to be significant (*p* < 0.0167).
