# Supplementary material for: Lytic Gene Expression Is Frequent in HSV-1 Latent Infection and Correlates with the Engagement of a Cell-Intrinsic Transcriptional Response
Source: PLoS Pathog. 2014 Jul 24;10(7):e1004237. doi: 10.1371/journal.ppat.1004237 (PMC4110040; doi:10.1371/journal.ppat.1004237)
Supplement: Table S4 — p values for the proportion of cells expressing the gene from comparisons within all, Ntrk1 + and LAT+ Ntrk1 +YFP+ neurons. (DOCX) [file ppat.1004237.s008.docx]

**Table S4.** *p* values for the proportion of cells expressing the gene from comparisons within all, *Ntrk1*^+^ and LAT^+^*Ntrk1*^+^YFP^+^ neurons.

|  | All neurons | | | *Ntrk1*^+^ neurons | | | LAT^+^*Ntrk1*^+^YFP^+^ neurons | | |
| --- | --- | --- | --- | --- | --- | --- | --- | --- | --- |
|  | YFP^+^ **vs.** YFP^-^ | YFP^+^ **vs.** Uninfected | YFP^-^ **vs.** Uninfected | YFP^+^ **vs**. YFP^-^ | YFP^+^ **vs**. Uninfected | YFP^-^ **vs**. Uninfected | Full-lytic **vs**. Partial-lytic | Full-lytic **vs**. non-lytic | Partial-lytic **vs**. non-lytic |
| *Pgm2l1* | **0.0170** | 0.1250 | 0.7220 | 0.5510 | 0.0590 | 0.3500 | **0.0430** | **0.0070** | 0.7270 |
| *Tbp* | 0.3340 | 0.6000 | 0.1510 | 0.3380 | 0.4320 | 0.1310 | 0.0790 | **0.0000** | 0.0970 |
| *Pias2* | 0.1710 | 0.7900 | 0.1240 | 0.1190 | 0.5050 | 0.0550 | 0.1590 | **0.0010** | 0.1160 |
| *Pias1* | 0.8580 | 0.2480 | 0.1740 | 0.3660 | 0.3240 | 0.1020 | 0.1290 | **0.0000** | 0.0740 |
| *Xrcc5* | 0.3040 | 0.0580 | 0.2960 | 0.8560 | 0.1240 | 0.2260 | 0.6580 | **0.0040** | 0.0740 |
| *Atr* | 0.1650 | 0.8910 | 0.2520 | **0.0250** | 0.6310 | **0.0170** | 0.1370 | **0.0410** | 0.7410 |
| *Samhd1* | 0.3420 | 0.4850 | 0.8750 | 0.0700 | 0.9450 | 0.1320 | 0.0790 | **0.0110** | 0.7280 |
| *Dicer1* | 0.9190 | 0.3650 | 0.3970 | 0.2080 | 0.7770 | 0.1840 | **0.0210** | **0.0040** | 0.6340 |
| *Ifit3* | **0.0110** | 0.1130 | 0.4780 | **0.0200** | 0.7440 | 0.0770 | 0.2160 | **0.0440** | 0.5800 |
| *Ifitm3* | **0.0000** | **0.0090** | 0.3640 | **0.0080** | 0.2630 | 0.1630 | **0.0080** | **0.0120** | 0.7410 |
| *Dhx36* | 0.2900 | 0.8790 | 0.2580 | 0.7070 | 0.4720 | 0.7790 | 0.6580 | 0.1700 | 0.7230 |
| *Dhx9* | 0.7570 | 0.9640 | 0.7400 | 0.7790 | 0.5870 | 0.8250 | 0.0600 | **0.0020** | 0.4000 |
| *Ddx58* | **0.0040** | **0.0010** | 0.3400 | **0.0150** | 0.1020 | 0.4700 | 0.4430 | **0.0210** | 0.3540 |
| *Mx1* | **0.0000** | **0.0000** | 0.1600 | **0.0000** | **0.0000** | 0.4810 | 0.8510 | **0.0160** | **0.0230** |
| *Eif2ak2* | **0.0300** | 0.2080 | 0.4690 | 0.1760 | 0.4210 | 0.6250 | 0.4430 | **0.0010** | **0.0470** |
| *Oasl2* | **0.0000** | **0.0000** | 0.2390 | **0.0000** | **0.0000** | 0.7580 | 0.1260 | **0.0030** | 0.2890 |
| *Oas1c* | 0.5430 | 0.9970 | 0.5750 | 0.4650 | 0.5630 | 0.2560 | 0.1430 | **0.0010** | 0.1520 |
| *Ifih1* | **0.0130** | **0.0240** | 0.9210 | **0.0410** | 0.1290 | 0.5900 | 0.1470 | 0.0990 | 1.0000 |
| *Isg20* | **0.0070** | 0.3300 | 0.1400 | 0.1000 | 0.4290 | 0.4310 | 0.1570 | **0.0000** | 0.0570 |
| *Aim2* | 0.7050 | 0.3900 | 1.0000 | 0.3040 | 0.6610 | 1.0000 | 1.0000 | 1.0000 | 1.0000 |
| *Ifit1* | **0.0000** | **0.0000** | 0.7110 | **0.0200** | **0.0120** | 0.9020 | 0.1770 | **0.0060** | 0.2690 |
| *Ifi204* | **0.0000** | **0.0000** | 1.0000 | **0.0020** | **0.0190** | 0.6190 | 0.5090 | 0.6100 | 0.7200 |
| *Tmem173* | 0.1730 | 0.5240 | 0.5510 | 0.2680 | 0.7850 | 0.4910 | 0.4050 | 0.9780 | 0.3840 |
| *Zbp1* | **0.0000** | **0.0000** | 0.0870 | **0.0000** | **0.0000** | 1.0000 | 0.0970 | 0.1380 | 0.7280 |
| *Apobec3* | 0.1220 | 0.0700 | 0.6130 | 0.8200 | 0.2630 | 0.2380 | 0.0610 | 0.1460 | 0.4540 |
| *Apobec1* | **0.0170** | **0.0100** | 0.5650 | 0.1800 | 0.1240 | 0.8790 | 0.6400 | 0.4950 | 0.8940 |
| *H2-T23* | **0.0000** | **0.0000** | 0.1940 | **0.0000** | **0.0010** | 0.5540 | **0.0070** | **0.0240** | 0.5090 |
| *Serpinb9* | **0.0010** | 0.1290 | 0.1230 | **0.0010** | 0.1360 | 0.0720 | **0.0280** | **0.0130** | 1.0000 |
| *Fadd* | 0.4960 | 0.4530 | 0.8740 | 0.1780 | 0.5690 | 0.4570 | **0.0180** | **0.0020** | 1.0000 |
| *Cflar* | **0.0030** | 0.1230 | 0.2190 | **0.0180** | 0.1780 | 0.3730 | 1.0000 | 0.3250 | 0.2890 |
| *Fas* | **0.0030** | **0.0150** | 1.0000 | **0.0050** | **0.0360** | 1.0000 | 0.7350 | 0.3260 | 0.6740 |
| *Tnfrsf10b* | **0.0000** | **0.0000** | 0.1660 | **0.0000** | **0.0000** | 0.9040 | **0.0000** | **0.0000** | 0.7160 |
| *Xiap* | 0.9090 | 0.2920 | 0.2300 | 0.4790 | 0.3080 | 0.7980 | 0.5470 | **0.0120** | 0.1160 |
| *Bax* | 0.8220 | 0.8130 | 0.7000 | 0.9250 | 0.5480 | 0.6590 | 0.2930 | **0.0200** | 0.2890 |
| *Bcl2l1* | 0.3060 | **0.0340** | 0.1900 | 0.4920 | 0.0770 | **0.0370** | 1.0000 | **0.0360** | 0.0700 |
| *Bcl2l11* | **0.0000** | **0.0270** | 0.1780 | **0.0410** | 0.2380 | 0.3900 | **0.0460** | **0.0010** | 0.4590 |
| *Bcl2* | **0.0420** | **0.0480** | 0.8630 | 0.3410 | **0.0380** | 0.3440 | **0.0120** | **0.0000** | 0.0970 |
| *Tnfrsf14* | **0.0000** | **0.0010** | 0.1810 | **0.0140** | **0.0070** | 0.5160 | **0.0050** | **0.0020** | 0.9070 |
| *Pvrl1* | **0.0000** | **0.0000** | 0.6000 | **0.0470** | **0.0010** | 0.2690 | 0.0590 | **0.0010** | 0.2160 |
| *Hcfc1* | 0.8740 | 0.9380 | 0.9470 | 0.0730 | 0.7200 | **0.0460** | 0.1260 | 0.0910 | 1.0000 |
| *Pou2f1* | 0.4400 | 0.4800 | 0.1470 | 0.1520 | 0.5520 | 0.0770 | 0.3780 | **0.0000** | **0.0070** |
| *Ntrk3* | **0.0000** | **0.0010** | 0.7720 | 0.9630 | **0.0240** | 0.0560 | 0.4980 | 0.1620 | 0.5800 |
| *Ntrk2* | 0.0570 | 0.5030 | 0.2840 | 0.6840 | 0.4160 | 0.7340 | **0.0130** | **0.0410** | 0.5030 |
| *Runx3* | 0.3480 | 0.1250 | 0.4170 | 0.5510 | 0.0590 | 0.3500 | 0.0790 | 0.7450 | 0.1410 |
| *Runx1* | **0.0000** | **0.0000** | **0.0020** | **0.0450** | **0.0000** | **0.0250** | 0.0510 | **0.0020** | 0.4180 |
| *Ret* | **0.0000** | **0.0000** | 0.7710 | **0.0020** | **0.0000** | 0.4770 | 0.1090 | **0.0120** | 0.5030 |
| *Ntrk1* | **0.0000** | **0.0000** | **0.0000** | Not tested | Not tested | Not tested | Not tested | Not tested | Not tested |
| *B2m* | 0.5200 | **0.0050** | **0.0230** | 0.5970 | **0.0480** | 0.4400 | - | 0.2070 | 0.5110 |
| *Pgk1* | 0.3800 | 0.6830 | 0.7000 | 0.8190 | 0.5600 | 0.4800 | 0.0520 | **0.0090** | 0.7440 |

**Bold** values are considered to be significant (*p* < 0.05).
