# Supplementary material for: Lytic Gene Expression Is Frequent in HSV-1 Latent Infection and Correlates with the Engagement of a Cell-Intrinsic Transcriptional Response
Source: PLoS Pathog. 2014 Jul 24;10(7):e1004237. doi: 10.1371/journal.ppat.1004237 (PMC4110040; doi:10.1371/journal.ppat.1004237)
Supplement: Table S9 — Taqman gene expression assays for cellular genes (Life Technologies). (DOCX) [file ppat.1004237.s013.docx]

**Table S9**. Taqman gene expression assays for cellular genes (Life Technologies)

| Gene name | Gene symbol | Gene aliases | Taqman Gene expression assay ID^1^ |
| --- | --- | --- | --- |
| RNA binding protein, fox-1 homolog (C. elegans) 3 | *Rbfox3* | NeuN | Mm01248771_m1 |
| Glial Fibrillary acidic protein | *Gfap* |  | Mm01253033_m1 |
| Neurotrophic tyrosine kinase, receptor, type 1 | *Ntrk1* | TrkA | Mm01219406_m1 |
| Neurotrophic tyrosine kinase, receptor, type 3 | *Ntrk3* | TrkC | Mm00456222_m1 |
| Neurotrophic tyrosine kinase, receptor, type 2 | *Ntrk2* | TrkB | Mm00435422_m1 |
| runt related transcription factor 3 | *Runx3* |  | Mm00490666_m1 |
| runt related transcription factor 1 | *Runx1* |  | Mm01213405_m1 |
| ret proto-oncogene | *Ret* |  | Mm00436304_m1 |
| Tumor necrosis factor receptor superfamily, member 14 | *Tnfrsf14* | HVEM | Mm00619239_m1 |
| Poliovirus receptor-related 1 | *Pvrl1* |  | Mm00445392_m1 |
| Host cell factor C1 | *Hcfc1* |  | Mm00468505_m1 |
| POU domain class 2 transcription factor 1 | *Pou2f1* | Oct1 | Mm00448332_m1 |
| Histocompatibility 2, T region locus 23 | *H2-T23* | Qa-1b | *Mm00439246_g1 |
| serine (for cysteine) peptidase inhibitor clade B, member 9 | *Serpinb9* |  | Mm00777163_m1 |
| Fas (TNFRSF6)-associated via death domain | *Fadd* |  | Mm00438861_m1 |
| CASP8 and FADD-like apopotsis regulator | *Cflar* | cFlip | Mm01255576_m1 |
| Fas (TNF receptor superfamily member 6) | *Fas* | CD95 | Mm01204974_m1 |
| Tumor necrosis factor receptor superfamily, member 10b | *Tnfrsf10b* | CD262, DR5 | Mm00457866_m1 |
| X-linked inhibitor of protein | *Xiap* |  | Mm00776505_m1 |
| Bcl2-associated X protein | *Bax* |  | Mm00432051_m1 |
| Bcl2-like 1 | *Bcl2l1* | BclXL | Mm00437783_m1 |
| Bcl2-like 11 (apoptosis faciliator) | *Bcl2l11* | Bim | Mm00437796_m1 |
| B-cell leukemia/lymphoma 2 | *Bcl2* |  | Mm00477631_m1 |
| X-ray repair complementing defective repair in Chinese hamster cells 5 | *Xrcc5* |  | Mm00550142_m1 |
| Ataxia telangiectasia and Rad3 related | *Atr* |  | Mm01223626_m1 |
| SAM domain and HD domain 1 | *Samhd1* |  | Mm00490121_m1 |
| Dicer1, Dcr-1 homolog (Drosophila) | *Dicer1* |  | Mm00521722_m1 |
| Interferon-induced protein with tetratricopeptide repeats 3 | *Ifit3* |  | *Mm01704846_s1 |
| Interferon induced transmembrane protein 3 | *Ifitm3* |  | *Mm03052142_s1 |
| DEAH box polypeptide 36 | *Dhx36* |  | Mm00661002_m1 |
| DEAH box polypeptide 9 | *Dhx9* |  | Mm00456021_m1 |
| DEAD (Asp-Glu-Ala-Asp) box polypeptide 58 | *Ddx58* |  | Mm01216853_m1 |
| Myxovirus resistance 1 | *Mx1* |  | Mm00487796_m1 |
| Eukaryotic translation initiation factor 2-alpha kinase 2 | *Eif2ak2* | PKR | Mm01235643_m1 |
| 2'-5' oligoadenylate synthetase-like 2 | *Oasl2* |  | Mm00496187_m1 |
| 2'-5' oligoadenylate synthetase 1C | *Oas1c* |  | Mm00459649_m1 |
| Interferon induced with helicase C domain 1 | *Ifih1* | Mda5 | Mm00459183_m1 |
| Interferon-stimulated protein | *Isg20* |  | Mm00469585_m1 |
| Absent in melanoma 2 | *Aim2* |  | Mm01295719_m1 |
| IFN-induced protein with tetratricopeptide repeats | *Ifit1* |  | Mm00515153_m1 |
| Interferon-inducible protein 16 | *Ifi204* | p204 | Mm00492602_m1 |
| Stimulator of interferon genes | *Tmem173* | STING | Mm01158117_m1 |
| Z-DNA binding protein 1 | *Zbp1* | DAI | Mm01247052_m1 |
| Apopliprotein B mRNA editing enzyme, catalytic polypeptide 3 | *Apobec3* |  | Mm01298575_m1 |
| Apopliprotein B mRNA editing enzyme, catalytic polypeptide 1 | *Apobec1* |  | Mm00482894_m1 |
| Protein inhibitor of activated STAT 2 | *Pias2* |  | Mm01257720_m1 |
| Protein inhibitor of activated STAT 1 | *Pias1* |  | Mm00497998_m1 |
| Beta-2 microglobulin | *B2m* |  | Mm00437762_m1 |
| Phosphoglycerate kinase 1 | *Pgk1* |  | *Mm00435617_m1 |
| TATA box binding protein | *Tbp* |  | Mm00446973_m1 |
| Phosphoglucomutase 2-like 1 | *Pgm2l1* |  | Mm00551045_m1 |

1. All assays use primers spanning exons except those marked with “*” where detection of genomic DNA cannot be excluded.
