# Supplementary material for: Lytic Gene Expression Is Frequent in HSV-1 Latent Infection and Correlates with the Engagement of a Cell-Intrinsic Transcriptional Response
Source: PLoS Pathog. 2014 Jul 24;10(7):e1004237. doi: 10.1371/journal.ppat.1004237 (PMC4110040; doi:10.1371/journal.ppat.1004237)
Supplement: Table S10 — Taqman probe and primers sequences for HSV-1 KOS genes. (DOCX) [file ppat.1004237.s014.docx]

**Table S10**. Taqman probe and primers sequences for HSV-1 KOS genes

| Viral gene name | Viral protein name | Class | Forward primer sequence | Reverse primer sequence | Taqman probe***** sequence | References |
| --- | --- | --- | --- | --- | --- | --- |
| *RL2* | ICP0 | IE | CGGACACGGAACTGTTCGA | CGCCCCCGCAACTG | CCCCATCCACGCCCTG | [[1](#_ENREF_1)] |
| *UL54* | ICP27 | IE | CGCCAAGAAAATTTCATCGAG | ACATCTTGCACCACGCCAG | CTGGCCTCCGCCGACGAGAC | [[2](#_ENREF_2)] |
| *US12* | ICP47 | IE | GTGCACGGCGGTTCTG | CGTACGCGATGAGATCAATAAAAGG | CCGCCTCCCGGTCCT | - |
| *UL39* | ICP6 | E | ATAGCCAATCCATGACCCTGTATG | GGGTGGAGGCTGGGAGG | CACGGAGAAGGCGGACGGGA | [[2](#_ENREF_2)] |
| *UL29* | ICP8 | E | CACCAGGTTGCGCATCAG | CTGCATACGGTGGTGAACAAC | ACCTCGCGGTCCACG | - |
| *UL27* | gB | L | CGCGAGCGTGTTGCT | GCAGTACTACCTGGCCAATGG | TCGCGTACCAGCCCC | - |
| *UL48* | VP16, ICP25 | L | TCGGGCCGCATCATCTG | GCGTGTTCTGTTTTTGCATTTGTAT | ACCCGCGAGATCCTAT | - |
| *US6* | gD | L | GCGGCTCGTGAAGATAAACG | CTCGGTGCTCCAGGATAAACTG | CTGGACGGAGATTACA | - |
| *UL44* | gC | L | GATGCCGGTTTCGGAATTC | CCCATGGAGTAACGCCATATCT | ACCCGCATGGAGTTCCGCCTC | [[2](#_ENREF_2)] |
| 2kb LAT | - | - | CGCCCCAGAGGCTAAGG | GGGCTGGTGTGCTGTAACA | CCACGCCACTCGCG | [[3](#_ENREF_3)] |

***** Taqman probe consists of a FAM dye label on the 5’ and minor groove binder (MGB) and non-fluorescent quencher (NFQ) on the 3’.

**Supplementary References**

1. Mott KR, Underhill D, Wechsler SL, Town T, Ghiasi H (2009) A role for the JAK-STAT1 pathway in blocking replication of HSV-1 in dendritic cells and macrophages. Virol J 6: 56.

2. Cohrs RJ, Randall J, Smith J, Gilden DH, Dabrowski C, et al. (2000) Analysis of individual human trigeminal ganglia for latent herpes simplex virus type 1 and varicella-zoster virus nucleic acids using real-time PCR. J Virol 74: 11464-11471.

3. Gussow AM, Giordani NV, Tran RK, Imai Y, Kwiatkowski DL, et al. (2006) Tissue-specific splicing of the herpes simplex virus type 1 latency-associated transcript (LAT) intron in LAT transgenic mice. J Virol 80: 9414-9423.
