# Supplementary material for: Correction: Lytic Gene Expression Is Frequent in HSV-1 Latent Infection and Correlates with the Engagement of a Cell-Intrinsic Transcriptional Response
Source: PLoS Pathog. 2014 Aug 8;10(8):e1004361. doi: 10.1371/journal.ppat.1004361 (PMC4126791; doi:10.1371/journal.ppat.1004361)
Supplement: File S2 — Republished corrected article. (PDF) [file ppat.1004361.s002.pdf]

OPEN ACCESS Freely available online

PLOS | PATHOGENS

Lytic Gene Expression Is Frequent in HSV-1 Latent Infection and Correlates with the Engagement of a Cell-Intrinsic Transcriptional Response

Joel Z. Ma[1]*, Tiffany A. Russell[2], Tim Spelman[3,4], Francis R. Carbone[1]*, David C. Tscharke[2]*

1 Department of Microbiology and Immunology, Peter Doherty Institute for Infection and Immunity, The University of Melbourne, Melbourne, Victoria, Australia, 2 Division of Biomedical Science and Biochemistry, Research School of Biology, The Australian National University, Canberra, Australian Capital Territory, Australia, 3 Victorian Infectious Diseases Service, Melbourne Health, Melbourne, Victoria, Australia, 4 Centre of Population Health, Burnet Institute, Melbourne, Victoria, Australia

Abstract

Herpes simplex viruses (HSV) are significant human pathogens that provide one of the best-described examples of viral latency and reactivation. HSV latency occurs in sensory neurons, being characterized by the absence of virus replication and only fragmentary evidence of protein production. In mouse models, HSV latency is especially stable but the detection of some lytic gene transcription and the ongoing presence of activated immune cells in latent ganglia have been used to suggest that this state is not entirely quiescent. Alternatively, these findings can be interpreted as signs of a low, but constant level of abortive reactivation punctuating otherwise silent latency. Using single cell analysis of transcription in mouse dorsal root ganglia, we reveal that HSV-1 latency is highly dynamic in the majority of neurons. Specifically, transcription from areas of the HSV genome associated with at least one viral lytic gene occurs in nearly two thirds of latently-infected neurons and more than half of these have RNA from more than one lytic gene locus. Further, bioinformatics analyses of host transcription showed that progressive appearance of these lytic transcripts correlated with alterations in expression of cellular genes. These data show for the first time that transcription consistent with lytic gene expression is a frequent event, taking place in the majority of HSV latently-infected neurons. Furthermore, this transcription is of biological significance in that it influences host gene expression. We suggest that the maintenance of HSV latency involves an active host response to frequent viral activity.

Citation: Ma JZ, Russell TA, Spelman T, Carbone FR, Tscharke DC (2014) Lytic Gene Expression Is Frequent in HSV-1 Latent Infection and Correlates with the Engagement of a Cell-Intrinsic Transcriptional Response. PLoS Pathog 10(7): e1004237. doi:10.1371/journal.ppat.1004237

Editor: Roger D. Everett, University of Glasgow, United Kingdom

Received January 23, 2014; **Accepted** May 23, 2014; **Published** July 24, 2014

Copyright: © 2014 Ma et al. This is an open-access article distributed under the terms of the Creative Commons Attribution License, which permits unrestricted use, distribution, and reproduction in any medium, provided the original author and source are credited.

Funding: This work was funded by NHMRC Project Grant APP1005846 (http://www.nhmrc.gov.au). The funders had no role in study design, data collection and analysis, decision to publish, or preparation of the manuscript.

Competing Interests: The authors have declared that no competing interests exist.

* Email: jzma@unimelb.edu.au (JZM); fcarbone@unimelb.edu.au(FRC); david.tscharke@anu.edu.au (DCT)

These authors contributed equally to this work.

Introduction

Herpes simplex virus (HSV) is a ubiquitous human pathogen that initially replicates in skin and mucosal surfaces prior to establishing an infection in sensory neurons [1]. There, HSV persists life-long with periods of prolonged quiescence punctuated by occasional episodes of reactivation, which may result in recrudescence of disease. As a consequence, HSV has long been viewed as establishing the prototypic latent infection, with minimal antigen expression and little to no active virus production during this phase of infection. While in humans, latency can be short-lived with multiple and frequent bouts of virus reactivation [2], in mice it is especially profound [3,4], making them the ideal model for detailed examination of this feature. Despite this general stability, one part of the HSV genome has long been known to be active in at least a fraction of neurons, giving rise to the expression of non-coding latency-associated transcripts (LAT) [5,6]. These transcripts have been postulated to play various roles in latency and this region is also the source of miRNAs that may also modulate infection [7–13]. In addition, there is sporadic, but consistent evidence that the transcription of genes normally associated with lytic replication also occurs in sensory ganglia during HSV latency, albeit at far lower levels than LAT [14–22]. There are competing explanations for the presence of these lytic transcripts. On the one hand, the lytic products may simply represent sporadic occurrences of complete reactivation, but at frequencies too low to permit ready detection of infectious virus or result in recrudescent disease [23]. An alternative proposal is that lytic transcripts are more widespread, found in a high proportion of infected cells during latency, but they represent abortive reactivation events that fail to progress to productive infection due to additional host control mechanisms [14,24]. Finally it is possible that these transcripts are simply the result of less than total silencing of the HSV genome, but are of no wider biological consequence.

The key to understanding the meaning of non-LAT transcription in HSV latency lies in the ability to examine events at the level of individual neurons. Recent advances in single-cell approaches to transcriptional analysis increase the sensitivity of detecting gene expression and allow the filtering out of samples containing transcripts from irrelevant cell types [25,26]. This means it is now possible to profile gene expression of individual cells using

Author Summary

Primary herpes simplex virus (HSV) infections are characterized by acute disease that resolves rapidly, but the virus persists in a latent form in sensory neurons that can be a source of renewed disease. Analyzing gene expression in single mouse neurons harboring latent HSV, we show directly that HSV latency is dynamic and heterogeneous. HSV lytic gene transcripts were frequently detected in latently infected neurons and often in combinations. Expression of selected cellular anti-viral and survival genes showed that transcriptional profiles differed between latently infected and uninfected neurons from the same ganglia. The pattern of host gene expression also differed between latently infected neurons that were and were not experiencing HSV lytic gene expression. Our study suggests that HSV latency is characterized by very frequent switching on of lytic genes and a rapid response by the host, presumably to halt progression to reactivation.

quantitative PCR, as reported in various studies [27–29]. One challenge in the application of these technologies to HSV latency is the identification and isolation of latently infected neurons from those that are uninfected and other cell types in sensory ganglia [30]. In this study, we exploited HSV-borne Cre recombinase to indelibly mark infected neurons in order to subject them to transcriptional analysis after laser capture microdissection (LCM) [31]. In doing so, we found that the presence of non-LAT viral transcripts, while at low levels, are detected very frequently in latently-infected neurons. Importantly, infected neurons respond to and match the viral activity with increasing host anti-viral gene expression, demonstrating that this viral transcription has biological consequences. These results demonstrate that the apparent stability of latency in virological terms hides a far more dynamic process in individual neurons.

Results

Presence of low level lytic transcripts during HSV latency in whole ganglia

Mice were inoculated by flank scarification using HSV type 1 (HSV-1) strain KOS, which results in the infection of the skin and innervating dorsal root ganglia (DRG) [32]. Here we define latency as a lack of virus production, and it is in place after the elimination of infectious virus in the DRG from around 7–8 days after inoculation (Figure 1A). We confirmed the presence of HSV DNA (Figure 1B) and 2 kb LAT intron (Figure 1C) in DRG at latency. Expression of lytic genes during latency has been reported by some [14–17], but not other studies [5,6,13,33–36] and so the meaning of the positive reports remains in question. Recent reviews tend to focus on the mechanisms by which lytic genes are silenced during latency, rather than questioning their possible importance [37,38]. Taking a conventional quantitative RT-PCR approach, we found LAT, but no transcripts consistent with lytic gene expression, during latency when whole DRG were examined (Figure 1C), in contrast to what was seen during the acute period of infection (days 50 and 5 post-inoculation, respectively). However, when a pre-amplification step was added to our protocol (see materials and methods), we were able to detect transcripts from all three tested lytic loci, namely *UL54* (ICP27), *UL29* (ICP8) and *US6* (gD) (Figure 1D).

In vivo labeled-infected neurons are numerically stable during latency

In order to move the analysis to single neurons, we employed a transgenic mouse model in which all infected neurons are marked so that they can be distinguished from other cells [39,40]. Transgenic mice having either yellow fluorescent protein (YFP) or β–galactosidase (*LacZ*) genes driven by the ubiquitous ROSA26 promoter interrupted with a floxed neomycin cassette were infected with HSVs expressing Cre recombinase under the CMV IE promoter (KOS0152 and KOS/pCMV/eGC, respectively) [40]. Transient expression of Cre occurs in every infected cell, regardless of fate, thereby leaving a legacy of marker gene expression that lasts for the life of the animal [39,40]. In our model, once the acute infection has subsided, DRG neurons marked in this way declined until day 20 and remained numerically stable until day 100, the latest time examined (Figure 2A). Others who have examined similar models in mice with the same methods have extended the observation of stable latency out to 147 days [39]. DRG from KOS0152-infected ROSA-YFP mice, but not mice infected with WT HSV or uninfected mice, contained fluorescence detectable by two-photon microscopy (Figure 2B) or in microscopy used for laser-dissection (Figure 2C) consistent with YFP expression in neurons. Samples examined by two-photon microscopy (Figure 2B) also showed an auto-fluorescent background consisting of scattered small bright particles. Thus, this method showed that once latency was established, the number of marked cells remained constant over a prolonged period and likely for the life of the infected mouse. More importantly, it provided us with a means of determining whether lytic transcripts could be detected in individual infected neurons and if so, determining the frequency and consequence of such expression for host gene expression.

Validation of a method for single cell analysis of transcription in HSV latency

Our approach to obtaining single neuron samples was to use laser capture microscopy (LCM) to identify by YFP expression and cut relevant cells from 8 μm sections of pooled latent DRG (Figure 3A). Marking of neurons in HSV-infected DRG was confirmed by co-localization of YFP expression with NeuroTrace staining, which detects Nissl bodies (Figure 3B, arrows). As a negative control, we never found YFP^+ neurons in DRG from uninfected mice. While the method worked well for the identification of latently-infected neurons, it had limitations; including the likelihood of partial cell capture, variations in capture volume, and inter-cell contamination. We reasoned that the impact of such limitations would be minimized if we examined many neuronal sections (hereafter referred to simply as neurons for brevity) in each analysis. In addition, variations associated with partial cell capture were expected to be consistent across all groups, allowing valid comparisons that would be similar to what could be found if whole neurons were to be used.

To test the reliability of the method, some preliminary studies were done. The first was to extract DNA from neurons and use qPCR to determine levels of HSV DNA in YFP^+ and YFP^- neurons. Most individual YFP^+ cells contained HSV DNA, whereas more than 80% of those with no detectable YFP did not (Table 1). The detection of HSV in YFP^- cells was most likely because of incomplete recombination of the ROSA-YFP locus, poor expression from the recombined genes or photo-bleaching of the YFP during capture. A proportion of YFP^+ cells did not show detectable levels of HSV DNA, probably because we were unable to detect fewer than 10 copies reliably, but also possibly due to

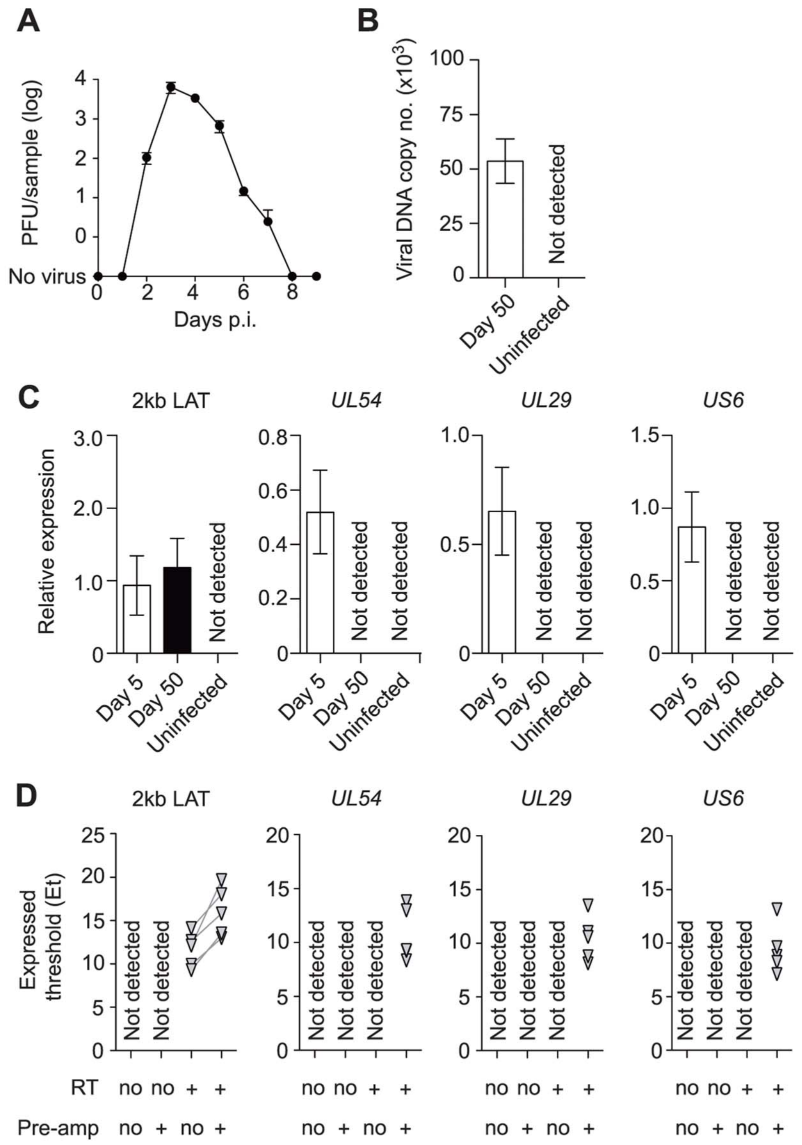

Figure 1. Infected DRG fulfill classical definitions of HSV latency. (**A**) Plaque forming units assay of HSV-infected DRG (T5 to L1) at various time points post inoculation. (**B**) Copy number of viral DNA from HSV-infected DRG (day 50) was determined by quantitative PCR. (**C**) Relative expression of HSV 2 kb LAT and lytic genes in HSV-infected DRG (days 5 and 50) were determined by quantitative PCR. (**D**) Expression (Et) of HSV 2 kb LAT and lytic genes in HSV-infected DRG (> day 240) were determined by quantitative PCR. Total RNA from each sample was aliquoted into four tubes and RT and pre-amplification were done as indicated beneath each graph. Pre-amplified products were used without dilution. Data in (**A–C**) are pooled from two independent experiments with 4–5 mice per group, (**D**) from one experiment with 5 mice, and plotted as mean ± S.E.M (**A–C**) or showing each individual result (**D**).
doi:10.1371/journal.ppat.1004237.g001

incomplete cell capture. Encouragingly, the distribution of HSV genome copy numbers per neuron above the detection threshold was consistent with previous studies [30,41]. The bulk of YFP$^+$ neurons contained fewer than 500 copies of viral DNA, with only a few containing more than 1000 copies (Figure 3C, Table 2).

To enable analysis of multiple transcripts on a single cell basis, we chose a highly sensitive method that committed each sample in its entirety to a single, random-primed reverse transcription (RT) followed by a multiplexed pre-amplification step before splitting into 61 qPCR (Taqman) reactions. This method has some caveats, for example we cannot verify the strand of transcripts (see discussion). This would also leave no material for controls, such as a sham RT. To ensure our extraction method was not frequently subject to DNA contamination, a set of 16 YFP$^+$ neurons was used in a test where each was split into two, enabling a sham RT control. We off-set the reduction in starting nucleic acid by using twice the usual amount of pre-amplified cDNA to quantify LAT. We reasoned that LAT should be detected in at least a proportion of neurons and because viral DNA contamination was the greatest concern. As shown as a heat map in Figure S1, LAT was detected in 15 out of 16 neurons by regular reactions, but none from the sham RT controls. This suggests that DNA contamination is infrequent if not absent in single neuron RNA samples prepared using our chosen method.

For the main analysis of transcription our study samples initially comprised RNA from 205 YFP$^+$ and 256 YFP$^-$ neurons from DRG of mice taken 50 days after infection (combined from four independent infections) and 136 neurons from DRG of uninfected

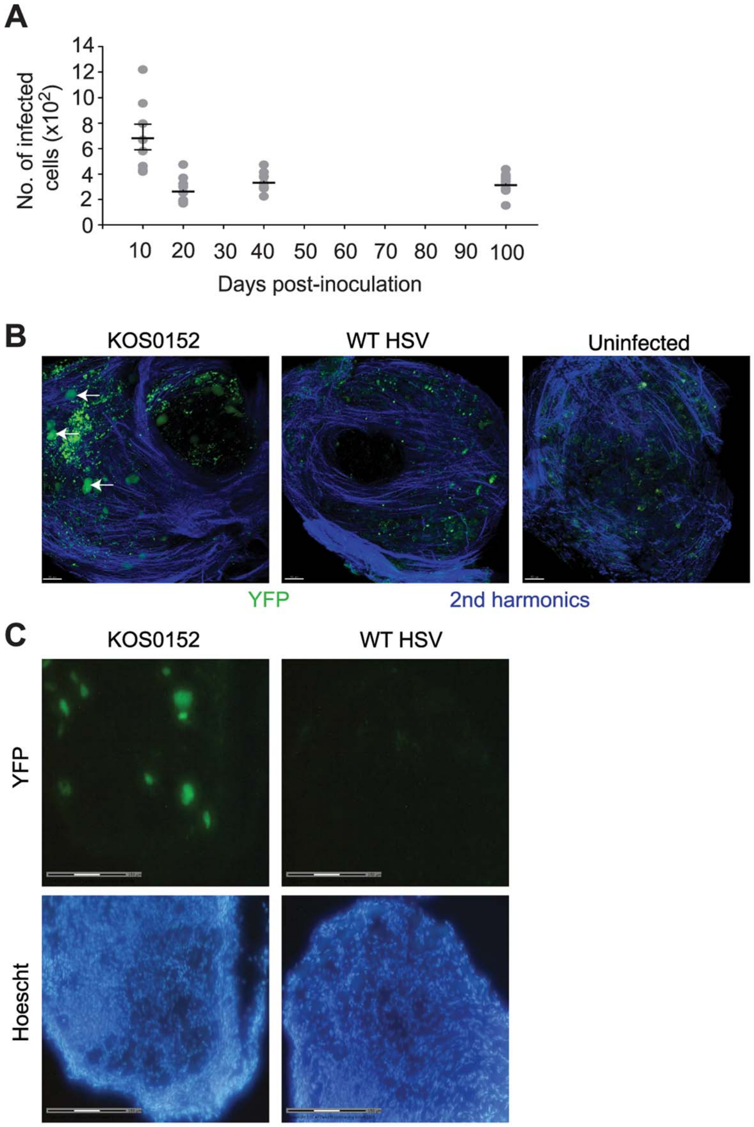

Figure 2. The number of infected cells remains stable during latency. (**A**) Number of infected (β-galactosidase$^+$) cells from KOS/pCMV/eGC infected DRG at the times shown post inoculation. (**B**) DRG explants from ROSA-YFP mice (day 20) infected with KOS0152 or WT HSV, or uninfected mice were examined under the 2 photon intra-vital microscope. Arrows identify HSV-infected cells. (**C**) DRG sections from KOS0152- or WT HSV-infected ROSA-YFP mice (day 50) were examined under the laser capture microscope for YFP expression and Hoechst nuclear stain. Data in (**A**) are pooled from two independent experiments and represented as mean ± S.E.M. Images in (**B** and **C**) are from two independent experiments.
doi:10.1371/journal.ppat.1004237.g002

mice (from three independent experiments). Each of these single cell RNA samples was analyzed with 61 primer/probe sets, targeting 51 cellular and 10 viral transcripts. Of the cellular transcripts, 47 of the 51 primers/probes spanned exon-exon boundaries. We included markers that allowed application of stringent selection criteria to neurons for further analyses. These included *Gfap* and *Rbfox3* as markers for satellite cells and neurons with adequate RNA, respectively. To eliminate the impact of possible errors in capturing neurons by LCM and spurious detections, only *Gfap*$^-$, *Rbfox3*$^+$ neurons in which more than 10% of the host genes could be detected were analysed further. In addition, uninfected neurons (YFP$^-$ or from uninfected DRG) in which any viral transcripts were detected were also excluded. Numbers of neurons excluded for any of these reasons and those remaining in analyses are noted in Table S1. The following analyses and results were all derived from this main data set.

Ntrk1$^+$ neurons harbor HSV during latency

Within the 51 cellular gene targets, 6 genes (*Ntrk1*, *Ntrk2*, *Ntrk3*, *Runx1*, *Runx3* and *Ret*) characterized different neuronal lineages. Analysis of these 6 genes showed that infection was heavily biased towards *Ntrk1*$^+$ neurons (Figure S2), since *Ntrk1* gene (encoding TrkA protein) expression was significantly over-represented in YFP$^+$ neurons: 86% of the YFP$^+$ neurons in the infected DRG were *Ntrk1*$^+$ compared to only 34% YFP$^-$ neurons (Figure 3D). Combined, the results demonstrated that YFP only marked infected neurons, predominantly of *Ntrk1*$^+$ peptidergic nociceptors [42]. Like the HSV DNA copy number, this result is consistent with the literature and suggests that our approach leads

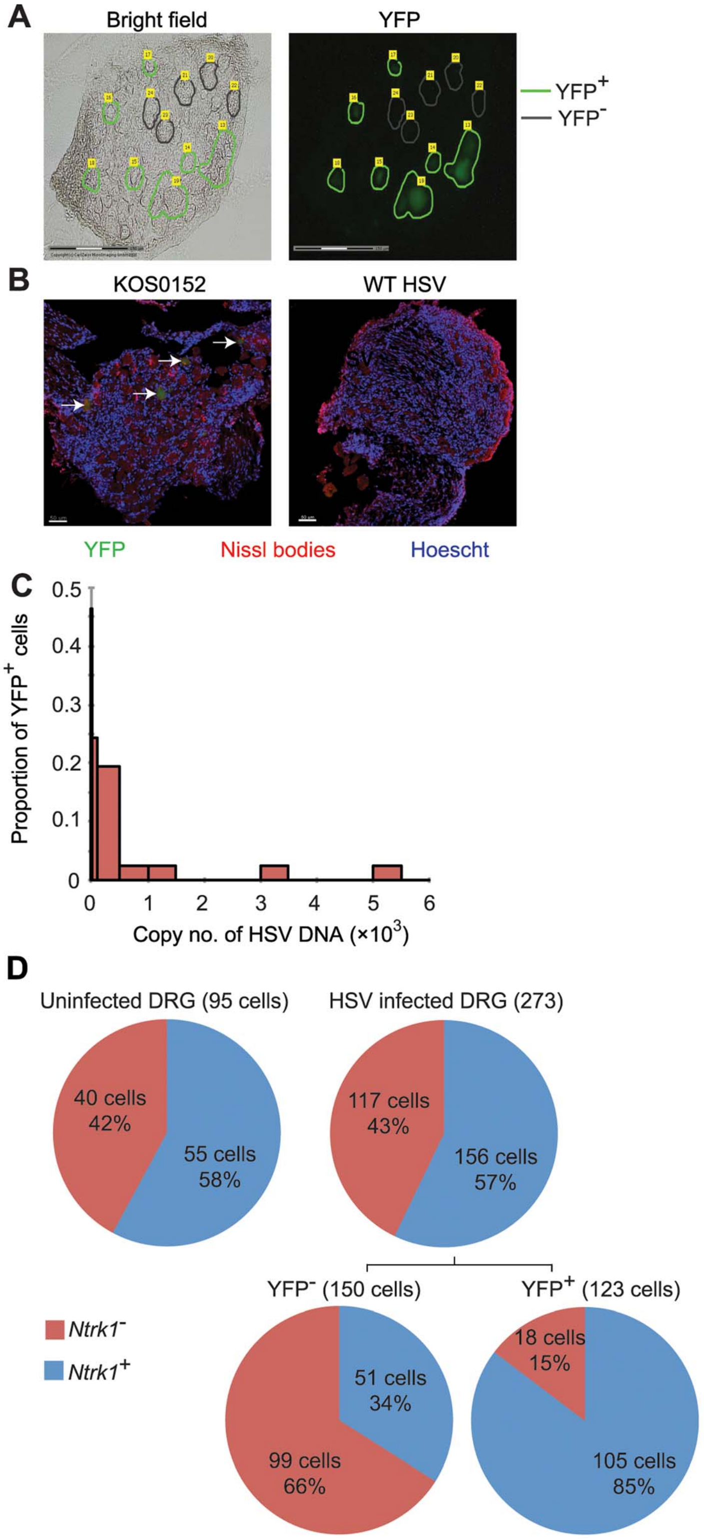

Figure 3. Latent HSV resides in a subpopulation of sensory neurons. (**A**) YFP$^+$ and YFP$^-$ cells were identified and captured from DRG sections of infected ROSA-YFP mice. (**B**) DRG sections from KOS0152- or WT HSV-infected ROSA-YFP mice (day 20) were stained for Nissl bodies and examined under the confocal microscope. Arrows identify HSV-infected neurons. (**C**) Copy number of viral DNA in individual YFP$^+$ cells (day 15) captured by LCM is shown in a histogram. (**D**) Number and percent of *Ntrk1*$^+$ and *Ntrk1*$^-$ neurons from populations of infected (YFP$^+$) and uninfected (YFP$^-$ and uninfected DRG) neurons in the main set of single cell expression data are shown in pie charts. Data in (**A** and **C**) are pooled from two independent experiments. Images in (**B**) are from one experiment. (**D**) As for all single cell gene expression data, the neuron sections analysed were combined from 4 independent infections (YFP$^+$ and YFP$^-$ neurons) or 3 independent experiments (neurons from uninfected DRG). Numbers in brackets show the number of individual cells analyzed.
doi:10.1371/journal.ppat.1004237.g003

Table 1. Number and percentage of YFP^+ and YFP^- cells containing HSV DNA.

	Number of cells		% cells of total cell number	
**HSV DNA**	**YFP^+**	**YFP^-**	**YFP^+**	**YFP^-**
Not detected	23	40	34.85	83.33
Detected	43	8	65.15	16.67
Total cell number captured	66	48		

doi:10.1371/journal.ppat.1004237.t001

to reliable data [43,44]. Further, this finding led us to narrow the rest of our overall analyses on *Ntrk1*$^+$ neurons to avoid finding transcriptional signatures associated with neuron subsets and not latency. At the same time, we note that *Ntrk1*$^+$ neurons are not homogenous and further single cell approaches may reveal details of HSV tropism within this population.

Frequent viral lytic gene expression in the majority of individual infected neurons during latency

Several studies comparing presence of HSV genomes by PCR-based methods and LAT expression in rodent sensory ganglia by conventional *in situ* hybridization found that only a subset of infected neurons expressed LAT during latency [41,45–47]. However, application of *in situ* RT-PCR suggests that LAT expression is more widespread [48] and this was also found in our RT controls (Figure S1). In the main data set, we found RNA consistent with 2 kb LAT in approximately 90% of latently-infected neurons (Figure 4A, 4B and 4D). Comparing this with the frequency of errors associated with our LCM method suggests strongly that all latently-infected neurons express LAT at levels detectable by our method. We therefore excluded YFP^+LAT^- samples from analyses to ensure that we were not including neurons that were not latently-infected.

Differences in sensitivity might also underlie variable accounts of lytic gene expression in latency (e.g. Figure 1C and D). We were able to detect transcripts consistent with lytic gene expression (called lytic transcripts hereafter, see discussion) in single cells and this was very frequent, occurring in 59% of all YFP^+ neurons (Figure 4C). When the analysis was narrowed to *Ntrk1*$^+$LAT$^+$ samples, which we can more confidently state are latently-infected neurons, the frequency rose to 64% (Figure 4E). Also of note was that many neurons had HSV transcripts from many lytic gene loci. HSV lytic genes are classified into 3 major kinetic classes, namely immediate early (IE), early (E) and late (L). To reduce the complexity in all the possible combinations of the lytic genes in our experiments, we assigned neurons to one of three groups: i) infected (YFP^+) neurons that did not express detectable lytic genes (non-lytic); ii) those that expressed lytic genes from 1 or 2, but not all 3, classes (partial-lytic); iii) those that expressed multiple gene transcripts with at least 1 member of each lytic class (full-lytic). Figure 4E shows that LAT^+YFP^+ neurons were roughly divided into thirds among the 3 categories. So there were roughly as many latently-infected neurons with transcripts consistent with the expression of lytic genes from all kinetic classes as there were that had none of these RNAs. Therefore even at times when latency appears to be highly stable, HSV-infected neurons express non-LAT transcripts at a frequency that has previously only been demonstrated to occur in lytic infection.

Infected neurons respond to latent virus by modulating host gene expression

It has been shown that at the level of the whole ganglia, latent infection alters transcriptional profiles, consistent with an antiviral response working to limit virus reactivation [49]. However, a study

Table 2. Number and proportion of YFP^+ cells containing various copy number of HSV DNA.

Copy number of HSV DNA		Number of YFP^+ cells	Proportion of YFP^+ cells
**Lower bound**	**Upper bound**		
0	10	19	0.463
10	100	10	0.244
100	500	8	0.195
500	1000	1	0.024
1000	1500	1	0.024
1500	2000	0	0.000
2000	2500	0	0.000
2500	3000	0	0.000
3000	3500	1	0.024
3500	4000	0	0.000
4000	4500	0	0.000
4500	5000	0	0.000
5000	5500	1	0.024

doi:10.1371/journal.ppat.1004237.t002

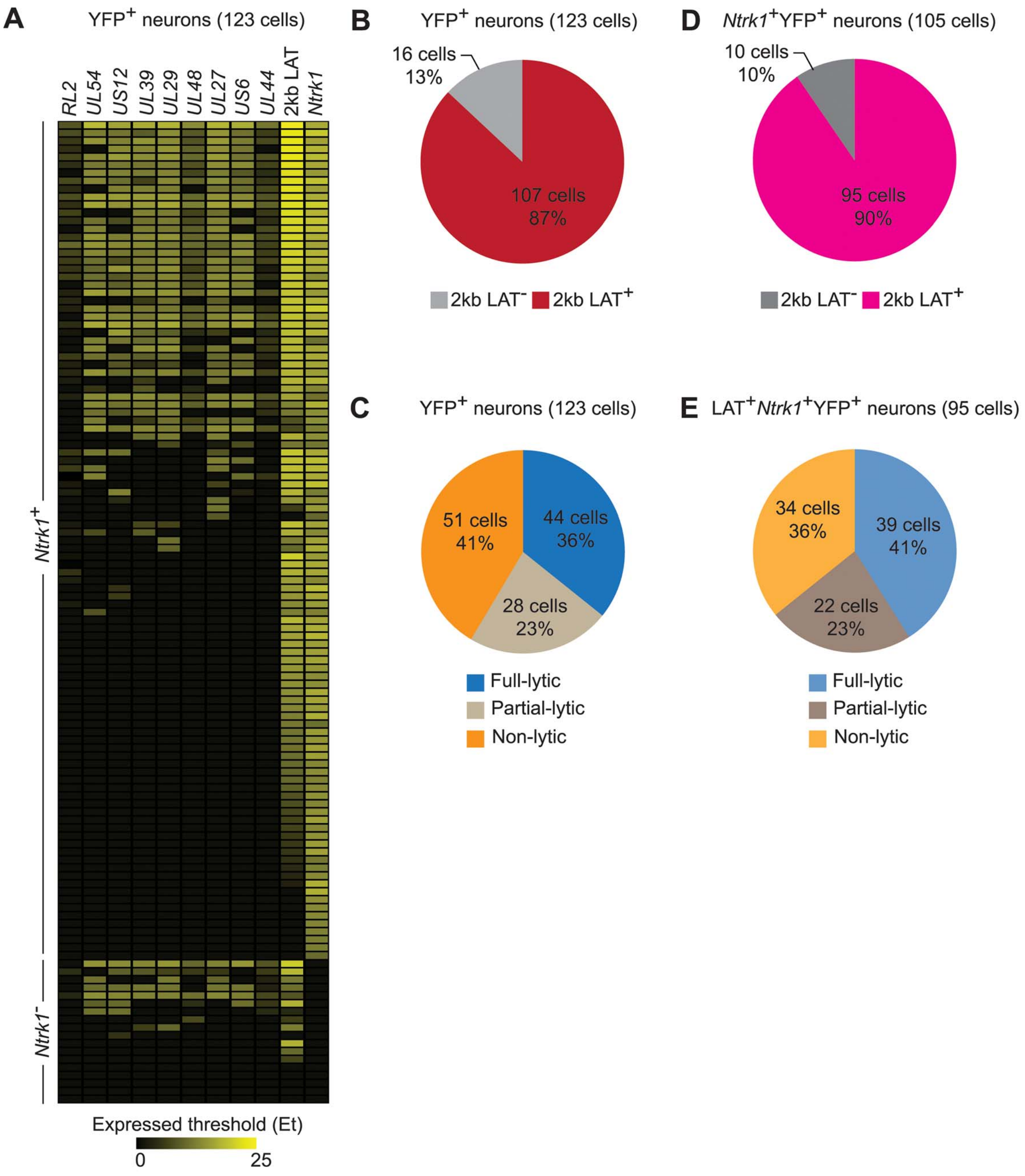

Figure 4. HSV gene expression in individual neurons during latency. (**A**) Heatmap showing HSV genes determined using quantitative RT-PCR in single YFP$^+$ neurons. (**B**) Number and percent of all infected YFP$^+$ neurons containing 2 kb LAT. (**C**) Number and percent of all infected YFP$^+$ neurons with different HSV lytic gene expression profiles. (**D and E**) As per B and C, but restricted to *Ntrk1*$^+$YFP$^+$ neurons. Numbers in brackets show the number of individual cells analyzed. All data are from the main single cell gene expression dataset.
doi:10.1371/journal.ppat.1004237.g004

of this design cannot dissect changes in neurons from those associated with other cell types, including immune infiltrates and it is possible that non-infected neurons within ganglia respond to infection in a bystander manner. These issues were addressed by an analysis of the anti-viral and cell-survival gene transcripts included in our qPCR panel. The processed gene expression data are shown as a heat map and the differences in the mean expression level between populations have been calculated (Figure S3 and Table S2). To reduce the complexity of this data, principal components (PC) analysis was used on 48 cellular genes in these *Ntrk1*$^+$ neurons. PC1, 2 and 3 captured 44.27%, 6.73% and 5.22% of the variation in the gene expression data, respectively. Most YFP$^+$ neurons segregated from those that were YFP$^-$ and neurons from uninfected DRG on PC2 and PC3, with some overlap captured on PC1 (Figure 5A). In contrast, YFP$^-$ neurons from infected mice and neurons from uninfected ganglia were relatively closely bunched together on PC1–3 (Figure 5B). Another way of looking at the differences between populations was to simply count the number of transcripts that differed both in level of expression and frequency of detection (Tables S3 and S4). In the case of infected and uninfected neurons from infected ganglia, 17 of the 48 genes differed. However, only a single transcript differed

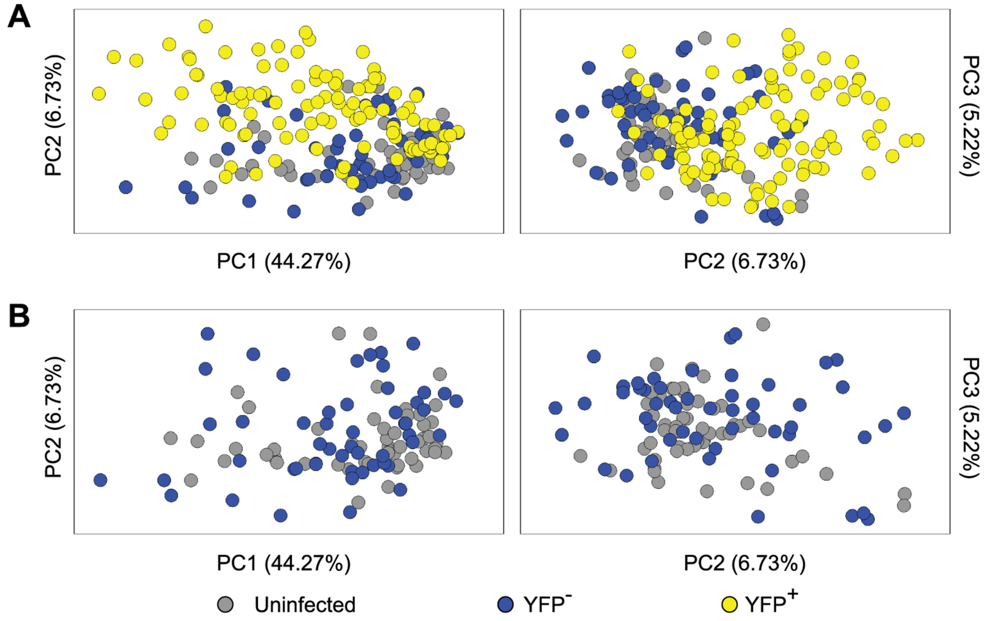

Figure 5. Single cell gene expression profiling reveals the transcriptional response of infected neurons towards latent HSV. (**A**) Principal components (PC) analysis of 48 cellular genes in single *Ntrk1*$^+$neurons – uninfected, YFP$^-$ and YFP$^+$. (**B**) PC analysis on *Ntrk1*$^+$YFP$^+$ neurons – YFP$^-$ versus uninfected. All data are from the main single cell gene expression dataset.
doi:10.1371/journal.ppat.1004237.g005

by both criteria between the two populations of uninfected neurons. These data show that HSV latency alters host gene expression in infected neurons, but do not provide strong evidence of transcriptional changes in non-infected neurons from infected ganglia. However, the latter observation may be a consequence of the limited set of genes examined.

Infected neurons match increasing viral activity by increasing anti-viral and survival gene expression

We reasoned that if the HSV lytic gene expression observed in latently-infected neurons was biologically relevant, it might influence the expression of host genes. For this reason, we compared host gene expression across the three groups of latently-infected neurons as identified by expression of HSV lytic genes (non-, partial- and full-lytic). To do this, we visualized the transcriptional data as violin plots (Figures 6A and S4). This representation is essentially a mirrored histogram that combines the transcript levels for each cellular gene (*y*-axis, represented by Log_2Ex) and the proportion of individual cells distributed along a given expression value (*x*-axis). The value of this representation can be seen in that most genes showed a bimodal distribution, indicating that the population contained individual cells with either high or low levels of a given gene transcript. We also calculated differences in mean transcript levels (Table S2) and whether level or frequency of detection was significantly different amongst the three populations of latently-infected neurons (Tables S3 and S4, respectively). We found that more than half (29/48) of the transcripts differed significantly both by level and frequency of detection between full- versus non-lytic groups, with fewer differences between full- versus partial- and partial- versus non-lytic neurons (8/48 and 1/48, respectively). Examples of some of these differences include: 1) *Oasl2*, *Mx1*, *Ddx58*, *SerpinB9*, *Bcl2l11*, *Pias1* and *Pvrl1* were expressed at significantly higher frequencies and levels in the full-lytic subgroup of neurons compared to the non-lytic subset. 2) Transcripts of *Tnfrsf10b*, *Bcl2* and *Tnfrsf14* were over-represented in full-lytic when compared to both of the other groups. 3) The single gene that differed between non-lytic and the groups that expressed lytic genes was *Pou2f1*. These data revealed heterogeneity in host gene expression in latently infected neurons that correlated with the number of classes of viral lytic genes detected. We conclude that transcription of HSV lytic genes during HSV latency is biologically relevant and suggest that even in the absence of overt reactivation, there is a progressive neuronal response against increasing viral activity.

Viral transcription activity alters neuronal transcriptional circuits during latency

Genes involved in the same biological processes or pathways are often expressed with correlated profiles. We wondered whether we could identify such sets of genes that were co-regulated in response to viral lytic gene transcription in latently-infected neurons. Finding these sets would provide evidence of changes to broader transcriptional circuits. To do this, we examined the correlations in expression profiles between pairs of cellular genes from full- and non-lytic neurons. In the full-lytic group, strong gene correlations ($\tau > 0.6$) were found between 75 pairs of genes (Tables S5 and S7). Importantly, the correlations were weaker and less frequent (only 23 pairs with $\tau > 0.6$) in the non-lytic group (Tables S6 and S8). Correlations did not only exist between pairs of genes but were also observed between 1 gene and several other genes. To show the co-dependencies between groups of genes, we clustered genes based on their correlation coefficients. First, we identified clusters of genes that were strongly correlated in full-lytic neurons and showed that these same clusters were less prominent in non-lytic neurons (Figure 6B, Table 3). Separately, we determined the optimal clustering of gene correlations in either full-lytic versus non-lytic neurons and this showed that viral lytic gene expression altered the optimal clustering patterns (Figure 6C). Thus, both comparisons found that sets of host transcripts and presumably the underlying regulatory mechanisms were altered in neurons where lytic gene loci were transcribed.

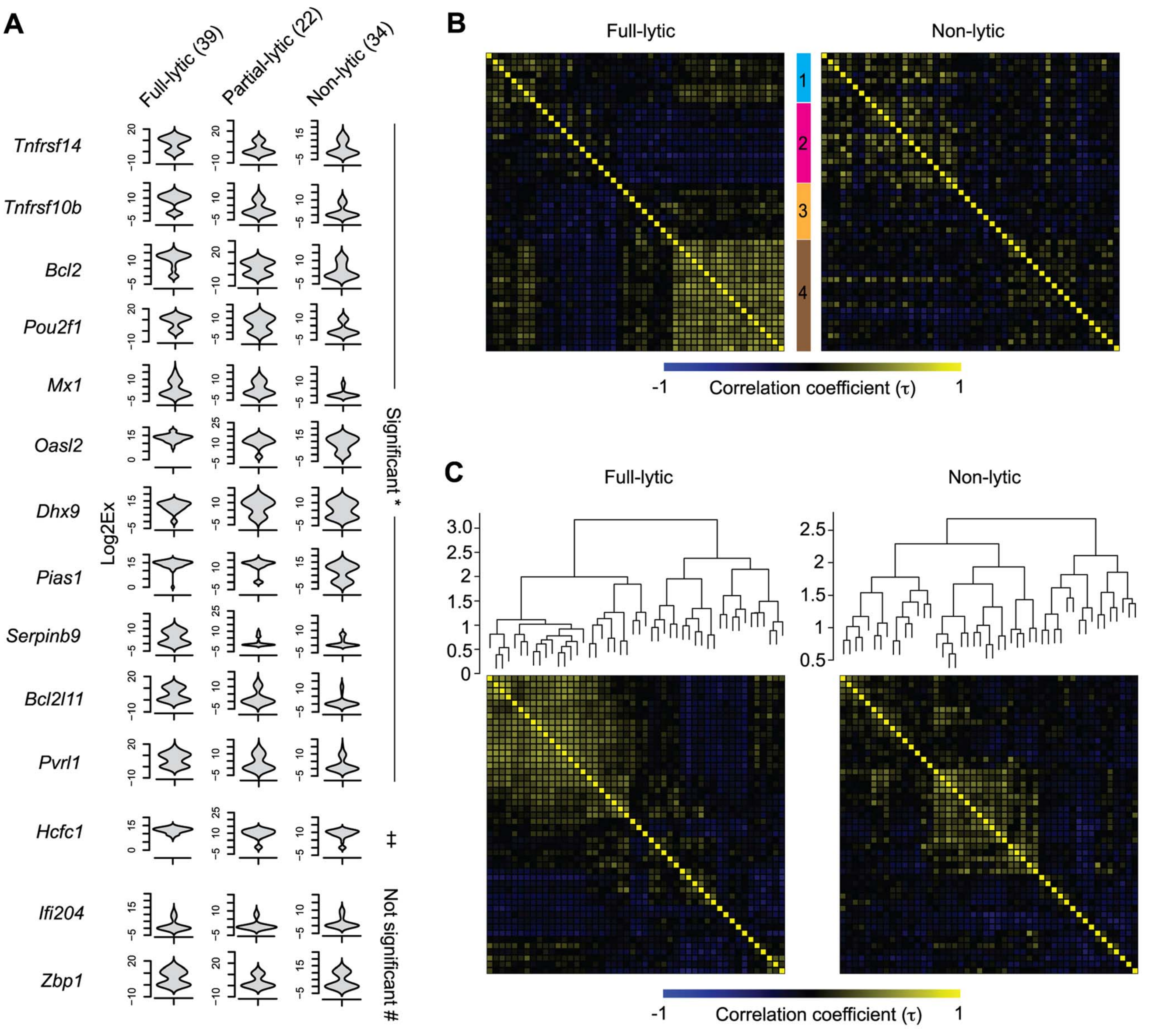

Figure 6. Increasing viral activity is matched by progressive host neuronal transcriptional response. (**A**) Violin plot representation of selected cellular gene expressions in LAT$^+$*Ntrk1*$^+$YFP$^+$ neurons categorized based on their lytic gene expression profile. Log2Ex represents expression threshold (Et). Numbers in brackets show the number of individual cells analyzed. * both proportion ($p<0.05$) and expression levels ($p<0.0167$) are significant, ‡ only expression levels significant, # proportion and expression levels are not significant when comparing full-lytic to partial- and non-lytic subsets. (**B**) *k*-means clustering of Kendall tau rank correlation coefficients (τ) of every pair of 48 genes from single LAT$^+$*Ntrk1*$^+$YFP$^+$ neurons. Correlation coefficient matrix of non-lytic neurons was clustered according to the optimal clustering observed in full-lytic neurons. (**C**) Complete-linkage clustering of Kendall tau rank correlation coefficients (τ) between expression profiles of every pair of 48 genes from single LAT$^+$*Ntrk1*$^+$YFP$^+$ neurons. Correlation coefficient matrices of non-lytic and full-lytic neurons were independently clustered. All data are from the main single cell gene expression dataset.
doi:10.1371/journal.ppat.1004237.g006

Discussion

Long-term HSV infection in mice is generally viewed as a model of profound latency, with few, if any, of the spontaneous reactivation episodes seen in other species and virtually no active production of virus once the lytic phase of infection is extinguished. In the context of this model of stable latency, the frequency with which transcripts consistent with lytic gene expression were found here is striking. Whilst our data does not break ground in terms of the detection of viral transcripts from outside the LAT region during latency, that this might happen so often has previously been only a matter of conjecture [14]. Perhaps more importantly, we also show that the host is equally active in responding to this viral activity, demonstrating that the viral transcription has biological consequences.

Some caveats remain in our study with regard to the nature of the viral transcripts detected. The most important of these is that we were unable to be certain that the transcripts detected were *bona fide* mRNAs from the various lytic genes. Some regions of the HSV genome are transcribed to produce co-linear messages for a set of genes and further, especially at late times of lytic infection, the termination of transcription is not always accurate.

Table 3. *k*-means clustering of gene expression correlation coefficients.

Cluster			
**1**	**2**	**3**	**4**
Ifitm3	*Ifit3*	*Fadd*	*Pgm2l1*
Ddx58	*Mx1*	*Tnfrsf10b*	*Tbp*
Oasl2	*Ifih1*	*Bcl2l11*	*Pias2*
Apobec3	*Isg20*	*Pvrl1*	*Pias1*
H2-T23	*Aim2*	*Pou2f1*	*Xrcc5*
Serpinb9	*Ifit1*	*Ntrk3*	*Atr*
Cflar	*Ifi204*	*Ntrk2*	*Samhd1*
B2m	*Tmem173*	*Runx1*	*Dicer1*
	Zbp1	*Ret*	*Dhx36*
	Apobec1		*Dhx9*
	Fas		*Eif2ak2*
	Tnfrsf14		*Oas1c*
	Runx3		*Xiap*
			Bax
			Bcl2l1
			Bcl2
			Hcfc1
			Pgk1

doi:10.1371/journal.ppat.1004237.t003

For this reason, in some cases we may not have detected only the HSV transcript intended. In addition, our reverse transcription method used random primers, being chosen to allow multiple transcripts to be detected at high sensitivity, but this also sacrifices strand-specificity. However, the nine HSV genes targeted are spread across the entire genome and different patterns of host gene expression are associated with increasing detection of the virus transcripts when ordered by expected kinetic classes. Furthermore, in some cases others have demonstrated elegantly that transcripts genuinely originating from specific HSV lytic genes are found in latently-infected ganglia [14]. Finally, if we are not detecting lytic gene expression, the alternative is that we have uncovered widespread random transcription of the HSV genome in latency that influences the host, which seems an unlikely proposition. Taking these together, the simplest explanation of our data is that we are detecting expression of HSV lytic genes, if perhaps not necessarily only those stated.

The relative roles of virus and host in maintaining latency remains an area of some contention, as is the extent to which detection of HSV transcripts other than LAT is of any significance. Much work has focused on mechanisms by which transcription from the majority of the genome is inhibited or regulated during latency, either through the silencing effect of heterochromatin or expression of miRNAs [1,7,37,50]. Alternatively, evidence of ongoing detection of HSV proteins by $CD8^+$ T cells [51–54] and the possibility that these can act in a non-lytic manner on neurons [32,55] suggest adaptive immunity might be an active player in the maintenance of latency. Our data do not bear on the first of these possibilities, except to demonstrate that the mechanisms that impede lytic gene transcription in latency substantially reduce, but do not silence this gene expression in the majority of neurons. The finding of frequent residual HSV lytic gene expression might be used to support a role for the adaptive immune system, but there remains the important caveat that we are only detecting RNA, with no evidence of translation. Such protein expression could be at an extremely low level, given the known sensitivity of T cells [56]. Also intriguingly, we found the expression of *Serpinb9*, which encodes a protein that can protect cells from $CD8^+$ T cell cytotoxicity, was up-regulated in the neurons showing the most viral transcription [57,58]. At the same time, we note that beyond the presence of activated T cells, evidence for viral protein expression in latency is conflicting. On the one hand, work showing that increased IE gene expression in viruses lacking LAT or miRNAs from the LAT region leads to increased transcription of genes from later classes might be interpreted to suggest that in these cases, IE protein in made [12,13,59]. On the other hand, these were not wild type viruses and a highly sensitive historical analysis based on expression of Cre from a variety of HSV lytic gene promoters that would allow low level or brief protein expression to be detected by Cre-reporter mice failed to detect any lytic gene expression [39].

These difficulties and our results could be used to argue for a third model in which the host acts to maintain latency in a cell-intrinsic manner. The viral transcription detected here may represent abortive reactivation events [14,24]. Of interest, we found significant expression of *Pou2f1* (Oct1), which is important for viral reactivation [60], in the full- and partial-lytic groups of neurons. We suggest these events fail to progress to productive infection due to detection of virus activity and a host response within neurons. This scenario requires that HSV activity in neurons is recognized by cell-intrinsic sensing mechanisms in the absence of translation of the lytic gene transcripts. Such mechanisms are well documented in cells other than neurons. In this regard, our finding that *Zbp1* (also referred to as DAI) is more frequently and significantly expressed in latently-infected, compared with uninfected neurons is of interest because this DNA sensor is associated with the response of fibroblasts to HSV [61]. The identity of the genes and possible mechanisms that might lead

to aborting reactivation remains obscure, but our data show significant up-regulation of several genes with established anti-viral roles in at least one relevant comparison (e.g. *Mx1*, *Oasl2*, *Eif2ak2* (PKR), *Ifitm3* and *Samhd1*). Thus, we propose that there is a barrier to productive infection downstream of the initiation of lytic gene transcription and this is a threshold that is rarely exceeded in mice [14]. Perhaps, understanding why the response to lytic gene transcription is so effective in this species, but not in people who suffer frequent reactivation and recurrence of disease, will highlight pathways of the greatest therapeutic potential. Finally, irrespective of the merits of any of these competing explanations, we would suggest that understanding the consequences of residual lytic gene expression will be important in any complete model of HSV latency.

Materials and Methods

Ethics statement

All experiments were done according to Australian NHMRC guidelines contained within the Australian Code of Practice for the Care and Use of Animals for Scientific Purposes and under approvals given by The University of Melbourne Animal Ethics Committee (1112345.7) and The Australian National University Animal Experimentation Ethics Committee (A2011/015).

Mice

C57BL/6 mice were bred at the Department of Microbiology and Immunology of the University of Melbourne. The gzmBCreERT2/ROSA26EYFP transgenic mice have been described previously [62]. gzmBCreERT2/ROSA26EYFP mice were not given tamoxifen, and will be referred to as ROSA-YFP. B6.129S4-Gt(ROSA)26Sor^{tm1So}/J, also referred to as ROSA26R mice, were from the Australian Phenomics Facility (ANU, Canberra).

Viruses and infections

The HSV-1 strains KOS0152, KOS/pCMV/eGC and wild-type parental strain KOS (WT HSV) were grown and titrated on Vero cells (CSL, Parkville, Australia) as described in [40]. KOS0152 contains the HCMV IE-Cre expression cassette inserted into the *UL43* gene of HSV-1. Similarly, KOS/pCMV/eGC contains HCMV IE promoter, but driving an EGFP-Cre fusion from the intergenic region beween *UL3* and *UL4*. Mice were infected with 1×10^6 PFU via flank scarification as previously described [63]. For single cell gene expression analysis, DRG from infected mice were collected day 50 post inoculation. Other time-points were as noted in the text and figure legends.

Laser capture microdissection

Mice were culled and tissues were fixed *in situ* by perfusing the mice from the left ventricle with 0.5% paraformaldehyde and 20% sucrose made in nuclease free PBS (fixative). DRG (T8–T12) were harvested, immersed in the fixative, and allowed to equilibrate on ice. Fixed DRG from two mice were pooled and then embedded in OCT (TissueTekIA018; Sakura) and frozen in liquid nitrogen. Frozen DRG were sectioned into 8 µm slices on a cryostat (Leica CM3050S) and consecutive DRG sections were placed on each subsequent slide (LOMB) that has been pre-treated with RNase-Away (Life Technologies). Freshly prepared tissue sections were dehydrated with 50%, 75% and 95% ethanol sequentially and air-dried just before laser capture. YFP$^-$ and YFP$^+$ cells were identified from 8–24 non-consecutive tissue sections and laser microdissected using a PALM MicroBeam instrument (Carl Zeiss). Laser capture was performed at the Biological Optical Microscopy Platform, University of Melbourne, Australia. Cells were then captured into collection tubes containing lysis buffer. We avoided collecting cells from 2–9 consecutive sections to prevent repeated sampling from the same cell. In total, 205 YFP$^+$ and 256 YFP$^-$ cells from HSV infected DRG and 136 cells from uninfected DRG were collected from 3–4 independent experiments with two mice per OCT block.

RNA and DNA extraction

Total RNA from LCM-captured single neurons was made into amplified cDNA using the Single Cell-to-CT kit (Life Technologies) according to manufacturer's instructions. Briefly, LCM-captured samples were lysed and treated with DNAse; and cell lysates were then reverse transcribed. In some samples, single cell lysates were split into halves whereby one half was reverse transcribed and the other was not. Products of RT (or sham RT) were then pre-amplified in a gene specific manner with a pool of 61 Taqman gene expression assays for 14 cycles; then diluted 1:15 to enable use in the same number of Taqman assays (Tables S9 and S10). Because 2 kb LAT is abundantly expressed in latency [1], we limited the 2 kb LAT primers and probe to a working concentration of 30 nM and 50 nM for pre-amplification, respectively. All other primers and probes were at 180 nM and 50 nM as per manufacturer's instructions, respectively.

Total RNA from whole DRG was extracted and DNAse-treated using RNAqueous microkit (Life Technologies) and reverse transcribed into cDNA using Superscript VILO (Life Technologies) that was then diluted 1:40. Aliquots of some samples were not reverse transcribed and served as 'no RT' controls. In some whole DRG samples, pre-amplification was carried out as described for single cell samples, but these pre-amplified products were not diluted (See Figure 1D).

DNA from LCM-captured neurons and whole DRG were extracted using the Picopure DNA extraction kit (Life Technologies). Briefly, single cells and DRG were incubated in proteinase K at 65°C for 3 h before enzyme inactivation at 95°C for 10 min.

Taqman quantitative PCR

Quantitative PCR assays were performed using Taqman gene expression assays (Tables S9 and S10) and Taqman Fast Advanced mastermix on the StepOnePlus real time PCR machine (Life Technologies). In single cell samples that were split into halves (to allow a matched sham RT control), the amount of pre-amplified cDNA used for quantitative PCR was doubled (See Figure S1). In some whole DRG analysis, viral gene expression was normalized to the housekeeping gene, *Pgk1*. Relative expression calculated using the $2^{-\Delta Ct}$ method [64] was used only to represent the absence and presence of viral transcripts.

HSV DNA detection in whole DRG and single neurons were performed as previously described [40]. The sequences, which are specific for the HSV thymidine kinase (*UL23*) gene, are as follows: forward primer 5′-TTGTCTCCTTCCGTGTTTCAGTT-3′, reverse primer 5′-GGCTCCATACCGACGATCTG-3′, and Taqman probe 5′-FAM-CCATCTCCCGGGCAAACGTGC-MGB-NFQ-3′. DNA extracted from uninfected DRG was used as negative control. Average viral copy number was calculated using a standard curve generated using WT HSV or KOS0152 viral DNA (extracted as described for DRG), which was serially diluted such that 5 µl contained 10^0 copies to 10^7 HSV-1 DNA. Our limit of detection for DNA was 10 copies, as we could not detect below 10 copies consistently. In single cell analysis, a duplex reaction containing the primers/FAM-probe for HSV TK gene,

and the primers/VIC-probe for β-actin (*Actb*) gene (Mm00607939_s1 VIC PL, Life Technologies) that detects genomic DNA was used to filter out LCM samples that did not contain any cells. In all single cell quantitative PCR, mock LCM samples, which contain lysis buffer but no cells, were used as negative controls.

Intravital two-photon microscopy

DRG were fixed *in situ* with 2% paraformaldehyde and 20% sucrose, harvested, secured onto a 10 mm dish with Vetbond tissue adhesive (3M), and immersed in PBS. Images were acquired with an upright LSM710 NLO multiphoton microscope (Carl Zeiss) as described previously [65]. EYFP was excited at 920 nm and collagen was visualized by second harmonic generation. Raw imaging data were processed with Imaris 7.1 (Biplane).

Histology, immunofluorescence and confocal microscopy

DRG from HSV infected ROSA26 mice were fixed and stained with X-Gal (BioVectra, Canada) as previously described [40]. DRG were then examined under light microscope (Olympus, Japan) and images of the DRG were taken with an Olympus DP20 camera. The number of β-galactosidase$^+$ cells was counted from the images with the aid of ImageJ software.

DRG were fixed *in situ* with and equilibrated in 2% paraformaldehyde and 20% sucrose before embedding in OCT and frozen in liquid nitrogen. DRG were sectioned at 8 µm thickness. Sections were fixed in −20°C acetone for 5 min, rehydrated with PBS for 5 min, stained with NeuroTrace (N-21482, Life Technologies) at 1:200 v/v in PBS for 20 min, rinsed in PBS as per manufacturer's instructions, incubated with Hoeschst nuclear stain (H33258, 1:3000 vol/vol in PBS) for 3 min, and then mounted with ProLongGold (P36934, Life Technologies). Images were acquired with a Zeiss LSM710 microscope and processed using Imaris 7.1 software (Bitplane).

Single-cell data processing

All Ct values obtained were converted into relative expression threshold (Et) by subtracting the values from the maximum cycle value of 40. When the value of the gene expression is undetermined, we treat this gene as not expressed and assigned an Et value '0' to the gene [66]. No normalization was performed for single cell analyses, which is the standard for this approach, because of large cell-to-cell variances in housekeeping gene expression [67,68] and because of the large biological sample size (e.g. ~200 neurons of each type). Cells that were negative for *Rbfox3* (neuron marker), were positive for *Gfap* (satellite cell marker), and expressed less than 10% of the 51 cellular genes tested were removed from analysis. We consider the remaining *Rbfox3*$^+$*Gfap*$^-$ cells as neurons. As noted above, transcription of genes follows intermittent bursts of biosynthesis [69], so some neurons may not express *Rbfox3* at the time of fixation and these would be wrongly excluded by our approach, but we felt that it was better to set conservative criteria for keeping samples in the final analyses. In a small fraction of YFP$^-$ neurons from infected DRG (27.4%) and uninfected neurons from uninfected DRG (8.5%), HSV transcripts were detected and these cells were excluded from subsequent analysis.

Single-cell data visualization

Principal components analysis was performed on 48 cellular genes using XLSTAT (Addinsoft SARL, US). Viral genes, *Rbfox3*, *Gfap* and *Ntrk1* were excluded because single cells were categorized based on these genes. Eigenvectors were then visualized with XLSTAT-3Dplot (Addinsoft SARL, US). Singular analysis toolset (Fluidigm, US) was used to generate violin plots [27]. Gene expression data and correlation coefficients were visualized as heatmaps using TM4 MultiExperimental Viewer [12].

Statistical analysis

Categorical variables were summarized using frequency and percentage. Continuous variables were first assessed for skew using a Shapiro-Wilk test and summarized using mean and standard deviation (SD) or median and inter-quartile range (IQR) as appropriate. Comparison of gene expression levels by neuron and lytic groups were undertaken using a Kruskal-Wallis test with a post-hoc Dunn test to correct for multiple simultaneous comparisons. As a sensitivity analysis, gene expression variables were dichotomized into expression recorded/no expression recorded quantities and compared across neuron or lytic groups using a chi-square test or a Fisher's exact test as appropriate. All analyses were conducted using Stata version 12 (StataCorp, College Station, Texas, US).

Correlation coefficients were calculated based on gene expression levels using Kendall rank correlation in XLSTAT (Addinsoft SARL, US). The correlation coefficients were then clustered using *k*-means or complete-linkage. For *k*-means clustering, 4 clusters were chosen from the full-lytic YFP$^+$ neurons subset based on the elbow method by comparing the sum of squared error (SSE) of a number of clusters. *k*-means clustering, complete-linkage clustering and SSE were performed in R statistical program.

Supporting Information

Figure S1 Single cells are free of contaminating viral DNA. Heatmap showing detection of 2 kb LAT in standard (+RT) and sham (No RT) reverse transcribed RNA from a set of 16 YFP$^+$ neurons.
(TIF)

Figure S2 Heatmap showing expression of neuronal-related genes in single neurons (*Rbfox3*$^+$*Gfap*$^-$) determined using quantitative RT-PCR. Data from main single cell experiments.
(TIF)

Figure S3 Heatmap showing 48 cellular genes in single *Ntrk1*$^+$ neurons determined using quantitative RT-PCR. Data from main single cell experiments.
(TIF)

Figure S4 Violin plot representation of 48 cellular transcripts in LAT$^+$*Ntrk1*$^+$YFP$^+$ neurons, categorized based on their lytic gene expression profiles. Data from main single cell experiments.
(TIF)

Table S1 Numbers of neurons excluded from gene transcripts analysis.
(DOCX)

Table S2 Ratio of mean gene expression levels (Et) from comparisons within all, *Ntrk1*$^+$ and LAT$^+$*Ntrk1*$^+$YFP$^+$ neurons.
(DOCX)

Table S3 *p* values for the gene expression levels from comparisons within all, *Ntrk1*$^+$ and LAT$^+$*Ntrk1*$^+$YFP$^+$ neurons.
(DOCX)

Table S4 *p* values for the proportion of cells expressing the gene from comparisons within all, *Ntrk1*$^+$ and LAT$^+$*Ntrk1*$^+$YFP$^+$ neurons.
(DOCX)

Table S5 Full-lytic LAT$^+$*Ntrk1*$^+$YFP$^+$ neurons: Complete-linkage clustering of gene expression correlation coefficients matrix.
(XLSX)

Table S6 Non-lytic LAT$^+$*Ntrk1*$^+$YFP$^+$ neurons: Complete-linkage clustering of gene expression correlation coefficients matrix.
(XLSX)

Table S7 Full-lytic LAT$^+$*Ntrk1*$^+$YFP$^+$ neurons: p values of complete-linkage clustering of gene expression correlation coefficients matrix.
(XLSX)

Table S8 Non-lytic LAT$^+$*Ntrk1*$^+$YFP$^+$ neurons: p values of complete-linkage clustering of gene expression correlation coefficients matrix.
(XLSX)

Table S9 Taqman gene expression assays for cellular genes (Life Technologies).
(DOCX)

Table S10 Taqman probe and primers sequences for HSV-1 KOS genes.
(DOCX)

Acknowledgments

We thank Alison Abendroth, Barry Slobedman, Claerwen Jones and Bethany Macleod for comments on the manuscript.

Author Contributions

Conceived and designed the experiments: DCT FRC JZM TAR. Performed the experiments: JZM TAR. Analyzed the data: DCT FRC JZM TS TAR. Contributed reagents/materials/analysis tools: TAR. Wrote the paper: DCT JZM FRC.

References

1. Jurak I, Silverstein LB, Sharma M, Coen DM (2012) Herpes Simplex Virus Is Equipped with RNA- and Protein-Based Mechanisms To Repress Expression of ATRX, an Effector of Intrinsic Immunity. J Virol 86: 10093–10102.
2. Wald A, Corey L, Cone R, Hobson A, Davis G, et al. (1997) Frequent genital herpes simplex virus 2 shedding in immunocompetent women. Effect of acyclovir treatment. J Clin Invest 99: 1092–1097.
3. Gebhardt BM, Halford WP (2005) Evidence that spontaneous reactivation of herpes virus does not occur in mice. Virol J 2: 67.
4. Mester JC, Rouse BT (1991) The mouse model and understanding immunity to herpes simplex virus. Rev Infect Dis 13 Suppl 11: S935–945.
5. Deatly AM, Spivack JG, Lavi E, Fraser NW (1987) RNA from an immediate early region of the type 1 herpes simplex virus genome is present in the trigeminal ganglia of latently infected mice. Proc Natl Acad Sci U S A 84: 3204–3208.
6. Stevens JG, Wagner EK, Devi-Rao GB, Cook ML, Feldman LT (1987) RNA complementary to a herpesvirus alpha gene mRNA is prominent in latently infected neurons. Science 235: 1056–1059.
7. Umbach JL, Kramer MF, Jurak I, Karnowski HW, Coen DM, et al. (2008) MicroRNAs expressed by herpes simplex virus 1 during latent infection regulate viral mRNAs. Nature 454: 780–783.
8. Peng W, Henderson G, Inman M, BenMohamed L, Perng GC, et al. (2005) The locus encompassing the latency-associated transcript of herpes simplex virus type 1 interferes with and delays interferon expression in productively infected neuroblastoma cells and trigeminal Ganglia of acutely infected mice. J Virol 79: 6162–6171.
9. Perng GC, Jones C, Ciacci-Zanella J, Stone M, Henderson G, et al. (2000) Virus-induced neuronal apoptosis blocked by the herpes simplex virus latency-associated transcript. Science 287: 1500–1503.
10. Thompson RL, Sawtell NM (2001) Herpes simplex virus type 1 latency-associated transcript gene promotes neuronal survival. J Virol 75: 6660–6675.
11. Mador N, Goldenberg D, Cohen O, Panet A, Steiner I (1998) Herpes simplex virus type 1 latency-associated transcripts suppress viral replication and reduce immediate-early gene mRNA levels in a neuronal cell line. J Virol 72: 5067–5075.
12. Chen SH, Kramer MF, Schaffer PA, Coen DM (1997) A viral function represses accumulation of transcripts from productive-cycle genes in mouse ganglia latently infected with herpes simplex virus. J Virol 71: 5878–5884.
13. Garber DA, Schaffer PA, Knipe DM (1997) A LAT-associated function reduces productive-cycle gene expression during acute infection of murine sensory neurons with herpes simplex virus type 1. J Virol 71: 5885–5893.
14. Kramer MF, Coen DM (1995) Quantification of transcripts from the ICP4 and thymidine kinase genes in mouse ganglia latently infected with herpes simplex virus. J Virol 69: 1389–1399.
15. Feldman LT, Ellison AR, Voytek CC, Yang L, Krause P, et al. (2002) Spontaneous molecular reactivation of herpes simplex virus type 1 latency in mice. Proc Natl Acad Sci U S A 99: 978–983.
16. Maillet S, Naas T, Crepin S, Roque-Afonso AM, Lafay F, et al. (2006) Herpes simplex virus type 1 latently infected neurons differentially express latency-associated and ICP0 transcripts. J Virol 80: 9310–9321.
17. Green MT, Courtney RJ, Dunkel EC (1981) Detection of an immediate early herpes simplex virus type 1 polypeptide in trigeminal ganglia from latently infected animals. Infect Immun 34: 987–992.
18. Chen SH, Lee LY, Garber DA, Schaffer PA, Knipe DM, et al. (2002) Neither LAT nor open reading frame P mutations increase expression of spliced or intron-containing ICP0 transcripts in mouse ganglia latently infected with herpes simplex virus. J Virol 76: 4764–4772.
19. Giordani NV, Neumann DM, Kwiatkowski DL, Bhattacharjee PS, McAnany PK, et al. (2008) During herpes simplex virus type 1 infection of rabbits, the ability to express the latency-associated transcript increases latent-phase transcription of lytic genes. J Virol 82: 6056–6060.
20. Kramer MF, Chen SH, Knipe DM, Coen DM (1998) Accumulation of viral transcripts and DNA during establishment of latency by herpes simplex virus. J Virol 72: 1177–1185.
21. Pesola JM, Zhu J, Knipe DM, Coen DM (2005) Herpes simplex virus 1 immediate-early and early gene expression during reactivation from latency under conditions that prevent infectious virus production. J Virol 79: 14516–14525.
22. Tal-Singer R, Lasner TM, Podrzucki W, Skokotas A, Leary JJ, et al. (1997) Gene expression during reactivation of herpes simplex virus type 1 from latency in the peripheral nervous system is different from that during lytic infection of tissue cultures. J Virol 71: 5268–5276.
23. Du T, Zhou G, Roizman B (2011) HSV-1 gene expression from reactivated ganglia is disordered and concurrent with suppression of latency-associated transcript and miRNAs. Proceedings of the National Academy of Sciences 108: 18820–18824.
24. Preston CM (2000) Repression of viral transcription during herpes simplex virus latency. J Gen Virol 81: 1–19.
25. Fritzsch FS, Dusny C, Frick O, Schmid A (2012) Single-cell analysis in biotechnology, systems biology, and biocatalysis. Annu Rev Chem Biomol Eng 3: 129–155.
26. Wang D, Bodovitz S (2010) Single cell analysis: the new frontier in 'omics'. Trends Biotechnol 28: 281–290.
27. Guo G, Huss M, Tong GQ, Wang C, Li Sun L, et al. (2010) Resolution of cell fate decisions revealed by single-cell gene expression analysis from zygote to blastocyst. Dev Cell 18: 675–685.
28. Narsinh KH, Sun N, Sanchez-Freire V, Lee AS, Almeida P, et al. (2011) Single cell transcriptional profiling reveals heterogeneity of human induced pluripotent stem cells. J Clin Invest 121: 1217–1221.
29. Dalerba P, Kalisky T, Sahoo D, Rajendran PS, Rothenberg ME, et al. (2011) Single-cell dissection of transcriptional heterogeneity in human colon tumors. Nat Biotechnol 29: 1120–1127.
30. Sawtell NM (1997) Comprehensive quantification of herpes simplex virus latency at the single-cell level. J Virol 71: 5423–5431.
31. Emmert-Buck MR, Bonner RF, Smith PD, Chuaqui RF, Zhuang Z, et al. (1996) Laser capture microdissection. Science 274: 998–1001.
32. Simmons A, Nash AA (1984) Zosteriform spread of herpes simplex virus as a model of recrudescence and its use to investigate the role of immune cells in prevention of recurrent disease. J Virol 52: 816–821.
33. Spivack JG, Fraser NW (1987) Detection of herpes simplex virus type 1 transcripts during latent infection in mice. J Virol 61: 3841–3847.
34. Steiner I, Mador N, Reibstein I, Spivack JG, Fraser NW (1994) Herpes simplex virus type 1 gene expression and reactivation of latent infection in the central nervous system. Neuropathol Appl Neurobiol 20: 253–260.
35. Krause PR, Croen KD, Straus SE, Ostrove JM (1988) Detection and preliminary characterization of herpes simplex virus type 1 transcripts in latently infected human trigeminal ganglia. J Virol 62: 4819–4823.
36. Wagner EK, Bloom DC (1997) Experimental investigation of herpes simplex virus latency. Clin Microbiol Rev 10: 419–443.
37. Knipe DM, Cliffe A (2008) Chromatin control of herpes simplex virus lytic and latent infection. Nat Rev Microbiol 6: 211–221.
38. Roizman B, Zhou G, Du T (2011) Checkpoints in productive and latent infections with herpes simplex virus 1: conceptualization of the issues. J Neurovirol 17: 512–517.
39. Proenca JT, Coleman HM, Connor V, Winton DJ, Efstathiou S (2008) A historical analysis of herpes simplex virus promoter activation in vivo reveals distinct populations of latently infected neurones. J Gen Virol 89: 2965–2974.

40. Wakim LM, Jones CM, Gebhardt T, Preston CM, Carbone FR (2008) CD8(+) T-cell attenuation of cutaneous herpes simplex virus infection reduces the average viral copy number of the ensuing latent infection. Immunol Cell Biol 86: 666–675.
41. Chen XP, Mata M, Kelley M, Glorioso JC, Fink DJ (2002) The relationship of herpes simplex virus latency associated transcript expression to genome copy number: a quantitative study using laser capture microdissection. J Neurovirol 8: 204–210.
42. Marmigere F, Ernfors P (2007) Specification and connectivity of neuronal subtypes in the sensory lineage. Nat Rev Neurosci 8: 114–127.
43. Margolis TP, Imai Y, Yang L, Vallas V, Krause PR (2007) Herpes simplex virus type 2 (HSV-2) establishes latent infection in a different population of ganglionic neurons than HSV-1: role of latency-associated transcripts. J Virol 81: 1872–1878.
44. Yang L, Voytek CC, Margolis TP (2000) Immunohistochemical analysis of primary sensory neurons latently infected with herpes simplex virus type 1. J Virol 74: 209–217.
45. Mehta A, Maggioncalda J, Bagasra O, Thikkavarapu S, Saikumari P, et al. (1995) In situ DNA PCR and RNA hybridization detection of herpes simplex virus sequences in trigeminal ganglia of latently infected mice. Virology 206: 633–640.
46. Maggioncalda J, Mehta A, Su YH, Fraser NW, Block TM (1996) Correlation between herpes simplex virus type 1 rate of reactivation from latent infection and the number of infected neurons in trigeminal ganglia. Virology 225: 72–81.
47. Ramakrishnan R, Levine M, Fink DJ (1994) PCR-based analysis of herpes simplex virus type 1 latency in the rat trigeminal ganglion established with a ribonucleotide reductase-deficient mutant. J Virol 68: 7083–7091.
48. Jurak I, Hackenberg M, Kim JY, Pesola JM, Everett RD, et al. (2014) Expression of Herpes Simplex Virus 1 MicroRNAs in Cell Culture Models of Quiescent and Latent Infection. J Virol 88: 2337–2339.
49. Jurak I, Kramer MF, Mellor JC, van Lint AL, Roth FP, et al. (2010) Numerous Conserved and Divergent MicroRNAs Expressed by Herpes Simplex Viruses 1 and 2. J Virol 84: 4659–4672.
50. Wang QY, Zhou C, Johnson KE, Colgrove RC, Coen DM, et al. (2005) Herpesviral latency-associated transcript gene promotes assembly of heterochromatin on viral lytic-gene promoters in latent infection. Proc Natl Acad Sci U S A 102: 16055–16059.
51. Khanna KM, Bonneau RH, Kinchington PR, Hendricks RL (2003) Herpes simplex virus-specific memory CD8+ T cells are selectively activated and retained in latently infected sensory ganglia. Immunity 18: 593–603.
52. van Lint AL, Kleinert L, Clarke SR, Stock A, Heath WR, et al. (2005) Latent infection with herpes simplex virus is associated with ongoing CD8+ T-cell stimulation by parenchymal cells within sensory ganglia. J Virol 79: 14843–14851.
53. van Velzen M, Jing L, Osterhaus AD, Sette A, Koelle DM, et al. (2013) Local CD4 and CD8 T-Cell Reactivity to HSV-1 Antigens Documents Broad Viral Protein Expression and Immune Competence in Latently Infected Human Trigeminal Ganglia. PLoS Pathog 9: e1003547.
54. Halford WP, Gebhardt BM, Carr DJ (1996) Persistent cytokine expression in trigeminal ganglion latently infected with herpes simplex virus type 1. J Immunol 157: 3542–3549.
55. Knickelbein JE, Khanna KM, Yee MB, Baty CJ, Kinchington PR, et al. (2008) Noncytotoxic lytic granule-mediated CD8+ T cell inhibition of HSV-1 reactivation from neuronal latency. Science 322: 268–271.
56. Davis MM, Krogsgaard M, Huse M, Huppa J, Lillemeier BF, et al. (2007) T cells as a self-referential, sensory organ. Annu Rev Immunol 25: 681–695.
57. Medema JP, Schuurhuis DH, Rea D, van Tongeren J, de Jong J, et al. (2001) Expression of the serpin serine protease inhibitor 6 protects dendritic cells from cytotoxic T lymphocyte-induced apoptosis: differential modulation by T helper type 1 and type 2 cells. J Exp Med 194: 657–667.
58. Zhang M, Park SM, Wang Y, Shah R, Liu N, et al. (2006) Serine protease inhibitor 6 protects cytotoxic T cells from self-inflicted injury by ensuring the integrity of cytotoxic granules. Immunity 24: 451–461.
59. Kramer MF, Jurak I, Pesola JM, Boissel S, Knipe DM, et al. (2011) Herpes simplex virus 1 microRNAs expressed abundantly during latent infection are not essential for latency in mouse trigeminal ganglia. Virology 417: 239–247.
60. Kim JY, Mandarino A, Chao MV, Mohr I, Wilson AC (2012) Transient reversal of episome silencing precedes VP16-dependent transcription during reactivation of latent HSV-1 in neurons. PLoS Pathog 8: e1002540.
61. Takaoka A, Wang Z, Choi MK, Yanai H, Negishi H, et al. (2007) DAI (DLM-1/ZBP1) is a cytosolic DNA sensor and an activator of innate immune response. Nature 448: 501–505.
62. Bannard O, Kraman M, Fearon DT (2009) Secondary replicative function of CD8+ T cells that had developed an effector phenotype. Science 323: 505–509.
63. van Lint A, Ayers M, Brooks AG, Coles RM, Heath WR, et al. (2004) Herpes simplex virus-specific CD8+ T cells can clear established lytic infections from skin and nerves and can partially limit the early spread of virus after cutaneous inoculation. J Immunol 172: 392–397.
64. Livak KJ, Schmittgen TD (2001) Analysis of relative gene expression data using real-time quantitative PCR and the 2(-Delta Delta C(T)) Method. Methods 25: 402–408.
65. Gebhardt T, Whitney PG, Zaid A, Mackay LK, Brooks AG, et al. (2011) Different patterns of peripheral migration by memory CD4+ and CD8+ T cells. Nature 477: 216–219.
66. McDavid A, Finak G, Chattopadyay PK, Dominguez M, Lamoreaux L, et al. (2013) Data exploration, quality control and testing in single-cell qPCR-based gene expression experiments. Bioinformatics 29: 461–467.
67. Warren L, Bryder D, Weissman IL, Quake SR (2006) Transcription factor profiling in individual hematopoietic progenitors by digital RT-PCR. Proc Natl Acad Sci U S A 103: 17807–17812.
68. Bengtsson M, Stahlberg A, Rorsman P, Kubista M (2005) Gene expression profiling in single cells from the pancreatic islets of Langerhans reveals lognormal distribution of mRNA levels. Genome Res 15: 1388–1392.
69. Raj A, Peskin CS, Tranchina D, Vargas DY, Tyagi S (2006) Stochastic mRNA synthesis in mammalian cells. PLoS Biol 4: e309.
